# Multi-user multi-objective computation offloading for medical image diagnosis

**DOI:** 10.7717/peerj-cs.1239

**Published:** 2023-03-08

**Authors:** Qi Liu, Zhao Tian, Guohua Zhao, Yong Cui, Yusong Lin

**Affiliations:** 1 School of Computer and Artificial Intelligence, Zhengzhou University, Zhengzhou, China; 2 Collaborative Innovation Center for Internet Healthcare, Zhengzhou University, Zhengzhou, China; 3 School of Cyber Science and Engineering, Zhengzhou University, Zhengzhou, China; 4 Department of Magnetic Resonance Imaging, The First Affiliated Hospital of Zhengzhou University, Zhengzhou, China; 5 School of Computer and Communication Engineering, Zhengzhou University of Light Industry, Zhengzhou, China; 6 Hanwei IoT Institute, Zhengzhou University, Zhengzhou, China

**Keywords:** Computation offloading, Risk awareness, Multi-objective, Prospect theory, Distributed optimization, Exact potential game

## Abstract

Computation offloading has effectively solved the problem of terminal devices computing resources limitation in hospitals by shifting the medical image diagnosis task to the edge servers for execution. Appropriate offloading strategies for diagnostic tasks are essential. However, the risk awareness of each user and the multiple expenses associated with processing tasks have been ignored in prior works. In this article, a multi-user multi-objective computation offloading for medical image diagnosis is proposed. First, the prospect theoretic utility function of each user is designed considering the delay, energy consumption, payment, and risk awareness. Second, the computation offloading problem including the above factors is defined as a distributed optimization problem, which with the goal of maximizing the utility of each user. The distributed optimization problem is then transformed into a non-cooperative game among the users. The exact potential game proves that the non-cooperative game has Nash equilibrium points. A low-complexity computation offloading algorithm based on best response dynamics finally is proposed. Detailed numerical experiments demonstrate the impact of different parameters and convergence in the algorithm on the utility function. The result shows that, compare with four benchmarks and four heuristic algorithms, the proposed algorithm in this article ensures a faster convergence speed and achieves only a 1.14% decrease in the utility value as the number of users increases.

## Introduction

Medical imaging examinations are currently required for over 70% of clinical diagnostic behaviors in hospitals (Jayashree & Bhuvaneswaran, 1970; Maglogiannis et al., 2017; Teng, Kong & Wang, 2019). However, medical data grows unusually fast with the advancement of information technology in smart medicine. There will be a total of 40 trillion GB of medical data in 2020, with 85–90% of that coming from medical imaging, which exacerbates the burden of doctors’ imaging diagnosis work. Doctors hope to use intelligent image diagnostic models (IIDM) for accelerating image diagnosis (Zivkovic et al., 2022), but the hospital personal computer configuration is not high enough to meet the demand.

A new hospital service architecture known as the medical imaging cloud has emerged (Lakshmi et al., 2021). It sends medical images to the IIDM in the central cloud for processing by utilizing cloud computing, big data, the Internet of Things, digital imaging technology, and Internet technology. Despite the rapid availability of diagnostic results, there are still questions. For instance, deep learning-based IIDM will generate various parameters, which will lead to a significant increase in computation. In addition, the extremely long-distance image transfer between the central cloud and the hospital takes up a huge amount of network bandwidth, resulting in large delay, energy consumption and communication overhead.

To overcome the above shortcomings, several researchers have recently found that computation offloading (Shakarami, Shahidinejad & Ghobaei-Arani, 2020, 2021; Xu et al., 2020; Tong et al., 2020; uz Zaman et al., 2021, 2022b) is a promising technology to solve this dilemma. Computation offloading, as one of the key technologies for edge computing (uz Zaman et al., 2022a), refers to the technology by which resource-constrained terminal devices (TDs) offload part or all of the computing tasks to the edge server execution. It comprises an offloading strategy and resource allocation (Li et al., 2020c). This article focuses on the former. Specifically, the diagnosis tasks of the TDs are first uploaded and then processed on the edge servers. Finally, the corresponding terminal device receives the results (Zhang et al., 2019b).

Many researchers have conducted extensive research on computation offloading. There are the following major issues, however, with the existing offloading works: (1) the edge server will provide resources to the terminal devices without charge. However, in a real communication and computing environment, the cost of computing resources and wireless communication is unavoidable. Doctors cannot enjoy edge server services for free, but have to pay a fee. (2) It is not possible to effectively integrate real environmental concerns into their decision-making. Although performing diagnostic tasks on the edge nodes decreases latency, it might increase energy consumption and payment. Therefore, it is important to propose a trade-off offloading model between latency, energy, and cost. (3) Users hold a risk-neutral attitude. However, it has been argued recently that users are risk awareness when using edge server’s resources, especially in a resource-constrained environments (Vamvakas, Tsiropoulou & Papavassiliou, 2019a). Specifically, users can be classified into aggressive and conservative according to their behavior characteristics. For aggressive users, they will exhibit risk-seeking behavior, who want to offload diagnosis tasks to edge servers to avoid using resources on terminal devices, even though edge servers may not provide data processing services for all users. Another type of conservative user exhibits risk-aversion behavior, who prefers to process diagnosis tasks on terminal devices. The reason is that the computing resources of edge server will be overused when multiple users use it simultaneously.

To be closer to the real communication and computing environment, therefore, when making the offloading decision of each user in the medical image cloud scenario, multiple factors are considered. The factors include the user’s risk awareness and a set of innovative objectives: delay, energy consumption and payment. Our ultimate optimization goal is to maximize the prospect theoretic utility of each user. To achieve this goal, we propose a multi-user multi-objective computation offloading for medical image diagnosis. First, we design the user’s utility function based on the Prospect Theory principle by combining the multi-objective of delay, energy consumption, and payment, which simulates the risk awareness behavior of each user during diagnosis task offloading. Second, to maximize the utility, the computation offloading problem first is expressed as a distributed optimization problem, then is transformed into a non-cooperative game among the users. Third, we prove that this game has Nash equilibrium (NE) points based on exact potential game and propose a low complexity computation offloading algorithm based on best response dynamics (BRD-CO) to reach an NE point. Finally, we conduct detailed simulation experiments. The results show that the BRD-CO algorithm can guarantee that each user has a higher prospect theoretic utility and a faster convergence speed when compared with four benchmarks and four heuristic algorithms.

Therefore, according to the above, this article proposes a computation offloading method that employs the following two element problems as a guide for investigation:

- The possibility of designing a more realistic optimization goal function based on user risk awareness and multiple objectives.
- The possibility of further improving the convergence speed of the offloading algorithm in a distributed manner.

Based on the system model constructed, the framework designed and the experimental results, the main contributions of this work are summarized as follows.

- We develop a more specific and detailed computation offloading model using the formal method. It more clearly reflects the execution process of the user’s diagnosis task on the edge server and terminal devices, respectively.
- We achieve a more realistic optimization goal. The multi-user and multi-objective computation offloading method are closer to the real world, which not only reflects the risk attitude of each user but also trade-off for delay, energy consumption and payment.
- We design a distributed offloading algorithm with a faster convergence speed. Each user wants more for computation and wireless communication resources during the execution of a diagnostic task, the computation offloading problem therefore is considered as a distributed optimization problem. We propose an optimal computation offloading algorithm based on best response dynamics, which requires only a few iterations to converge to the Nash equilibrium point.
- We have achieved a higher prospect theoretic utility. We implement the proposed BRD-CO algorithm and conduct detailed studies. The experimental results show that the proposed algorithm has statistical superiority and provides a higher prospect theoretic utility.

The rest of this article is organized as follows. “Related work” presents the related work. “Computation offloading system model” illustrates the computation offloading model and discusses the delay, energy consumption and payment under different offloading modes. “Multi-user multi-objective computation offloading for medical image diagnosis” introduces a multi-user multi-objective computation offloading for medical image diagnosis. “Numerical results” designs the simulation experiment and presents the numerical results. Finally, a summary of our work and future plan is presented in “Conclusions”.

## Related work

Massive medical image data is becoming more challenging to process and manipulate as the advancement of medical information (Zhang et al., 2017b). As a way of managing and procedure big data, cloud computing plays an important role (El-Seoud et al., 2017; Rahman, Khalil & Yi, 2019). Zhang et al. (2020) proposed a normal distribution splitting-based method for executing plenty of medical data parallel. On the other hand, we can use the parallel computing and data distribution functions of related systems, such as the MapReduce model and Hadoop model (Khezr & Navimipour, 2017; Mo, 2019; Duan, Edwards & Dwivedi, 2019). Based on the Hadoop, MapReduce and Spark, the researcher uses machine learning to predict and analyze the future complications of diabetic patients, which improved processing speed (Vineetha & Nandhana, 2022). In the framework of medical imaging cloud based on cloud computing, however, the distance between the central cloud and the hospital is so far that the transmission will consume a large amount of bandwidth and cause huge latency.

Recently, computation offloading has received more and more attention as one of the most promising solutions to this issue, and various offloading strategies have been proposed (Mao, Zhang & Letaief, 2016; Zhang et al., 2017a, 2019a; Guo, Li & Guan, 2019; Li et al., 2019b, 2020b, 2020a; Messous et al., 2019; Meng et al., 2019; Mitsis, Tsiropoulou & Papavassiliou, 2020; Zhu et al., 2020a, 2020b; Alioua et al., 2020; Tang & Wong, 2022; Wang et al., 2021; Chen & Liu, 2021). The differences between various computation offloading methods are shown in Table 1. There are currently only a few studies on accelerating the processing of medical image data by computation offloading, mostly focusing on areas such as the internet of vehicle, unmanned aerial vehicles, *etc*. On the grounds of the optimization goal, these strategies can be divided into four categories: reducing delay, reducing energy consumption (EC), balancing delay with energy consumption, and maximizing utility.

**Table 1:** Comparison of different computational offloading model.

References	Utilized technique	Performance metrics	Evaluation tools	Case study	Advantages	Disadvantages
Meng et al. (2019)	Markov decision process Learning approach	Delay	Simulation (NA)	Machine translation	Single server single user Multiserver multiuser Dynamic instantaneous rate estimation	High complexity Only the delay is considered
Mao, Zhang & Letaief (2016)	Lyapunov optimization	Delay	Simulation (NA)	Mobile apps	Decisions depend only on the current system state	Only the delay is considered
Zhu et al. (2020a)	Deep Reinforcement Learning	Delay	Simulation (python)	Internet of vehicles	Multiagent Distrubuted offloading decision making	Only the delay is considered
Tang & Wong (2022)	Deep Reinforcement Learning	Delay	Simulation (NA)	Mobile apps	Distributed offloading decision making	Only the delay is considered
Wang et al. (2021)	Deep Reinforcement Learning Convex optimization	Energy consumption	Simulation (python)	Unmanned aerial vehicle	Fast acquisition of UAV trajectory Low complexity matching algorithm	Only the energy consumption is considered
Zhang et al. (2019a)	Lyapunov optimization	Energy consumption	Simulation (matlab)	Mobile apps	Ensure high network stability	Only the energy consumption is considered
Chen & Liu (2021)	Deep Reinforcement Learning	Energy consumption	Simulation (NA)	Augmented reality	Multiagent	Only the energy consumption is considered
Li et al. (2020b)	Convex approximation	Energy consumption	Simulation (NA)	Unmanned aerial vehicle	An on-demand offloading service in emergency scenarios	Only the energy consumption is considered
Zhu et al. (2020b)	Convex optimization	Delay Energy consumption	Simulation (NA)	Unmanned aerial vehicle	Multiserver multiuser cooperative offloading algorithm	Considerations should be more comprehensive
Guo, Li & Guan (2019)	Lyapunov optimization	Delay Energy consumption	Simulation (matable & C++)	Internet of things	Dynamic computation requests	Considerations should be more comprehensive
Zhang et al. (2017a)	Artificial fish swarm algorithm	Delay Energy consumption	Simulation (NA)	Small cell networks	Multiserver multiuser The fronthual and backhual links are joint considered	Considerations should be more comprehensive
Li et al. (2019b)	Nonlinear programming queue theory	Delay Energy consumption	Simulation (matlab)	Internet of things	tradeoff between delay and energy consumption	Considerations should be more comprehensive
Messous et al. (2019)	Non-cooperative game	Utility	Simulation (NA)	Unmanned aerial vehicle	Distributed offloading decision making	Not considered a dynamic selection of the weighting parameters in utility function
Alioua et al. (2020)	sequential game	Utility	Simulation (C++)	Unmanned aerial vehicle	Cooperative offloading mechanism	Comprehensive network parameters is not considered
Li et al. (2020a)	0–1 programming	Utility	Simulation (NA)	Internet of Vehicles	Vehicle mobility Offloading tasks simultaneously through multicast	Energy consumption is not considered
Mitsis, Tsiropoulou & Papavassiliou (2020)	Non-cooperative game Prospect theory	Utility	Simulation (python)	Unmanned aerial vehicle	Risk awareness of user	Energy consumption and payment is not considered

Mao, Zhang & Letaief (2016), Meng et al. (2019), Zhu et al. (2020a), Tang & Wong (2022) are offloading strategies to reduce the delay. For instance, Mao, Zhang & Letaief (2016) proposed a dynamic offloading method based on Lyapunov optimization, considering the execution latency and task failure, which can decrease the task time by 64%. However, these offloading strategies are only designed to minimize the overall delay, without considering the potential energy consumption.

Zhang et al. (2019a), Li et al. (2020b), Wang et al. (2021), Chen & Liu (2021) are offloading strategies to reduce energy consumption. For instance, Wang et al. (2021) proposed a trajectory control algorithm based on convex optimization and deep reinforcement learning by combining the motion trajectory, user association, and resource allocation of UAVs. Similar to the previous optimization goal, these offloading strategies are only effective in reducing the overall energy consumption of the task. However, in some systems, users prefer to achieve relative stability between delay and energy consumption.

Zhang et al. (2017a), Guo, Li & Guan (2019), Li et al. (2019b), Zhu et al. (2020b) are offloading strategies to balance delay and energy consumption. For instance, Zhang et al. (2017a) introduced predation behavior, swarm behavior and following behavior into the artificial fish swarm algorithm, which saves 30% energy consumption. While these offloading strategies achieve a tradeoff between latency and energy consumption, they may not be applicable to all systems. The reason for this is that each system has different performance requirements, not all of which are latency and energy consumption.

Messous et al. (2019), Mitsis, Tsiropoulou & Papavassiliou (2020), Alioua et al. (2020), Li et al. (2020a) are offloading strategies to maximizing utility. For instance, Messous et al. (2019) used game-theoretical to reach a balance of energy consumption, delay and payment. Similarly, Alioua et al. (2020) also proposed a sequential game-based computation offloading strategy. Li et al. (2020a) proposed an algorithm jointly optimizing the delay and payment for task offloading. Mitsis, Tsiropoulou & Papavassiliou (2020) proposed a resource-based pricing and user’s risk awareness computation offloading scheme. The above-mentioned offloading strategies have been widely concerned because they can design different utility functions according to different scenarios, and create an appropriate offloading strategy to meet the needs of users.

In the view of prior works, few studies conjointly consider risk awareness, delay, energy consumption, and payment. Most of which focus on two or three aspects, and assumes that the risk-neutral behavior of the users in the process of task offloading. To simulate the resource consumption in the real-world environment, in this article, we propose a computation offloading model for maximizing the prospect theoretic utility of each user, which jointly considers: (1) a clearer formal description for the computation offloading model; (2) more realistic optimization goals; (3) a distributed offloading algorithm with a faster convergence speed; (4) higher prospect theoretic utility. We also conduct experiments to evaluate the BRD-CO algorithm under various parameters.

## Computation offloading system model

The scenario in this article is a medical image diagnosis in a medical image cloud. In this section, we construct a computation offloading system model and introduce three offloading modes.

### Notation description

For readability, Table 2 summarizes the notation used in this article.

**Table 2:** Notations.

Notation	Description	Notation	Description	Notation	Description
$TD$	The finite set of terminal devices	$t{d_i}$	The device used by the i-th user	$B$	The finite set of diagnosis tasks
${b_{t{d_i}}}$	The computation task of $t{d_i}$	$\mu$	The finite set of offloading proportions	${\mu _{t{d_i}}}$	The offloading proportion of $t{d_i}$
$\phi$	Task computational complexity	$\xi$	The expected profits	$P{U_0}$	The user’s anticipated profit
$P{U_{t{d_i}}}$	The user’s actual profit	${k_{t{d_i}}}$	The loss aversion coefficient	${\lambda _1}$	Delay weight
${\lambda _2}$	Energy consumption weight	${\lambda _3}$	Payment weight	$\omega$	Payment factor
$Pr$	The failure probability of the edge server	$\gamma$	Gain attitude	$\delta$	Loss attitude
$par{a^L}$	The finite set of local computing parameters	${F^L}$	The finite set of the computational capability	$f_{t{d_i}}^L$	The computational capability of $t{d_i}$
${\chi ^L}$	The finite set of the energy coefficient	$\chi _{t{d_i}}^L$	The consumed energy per CPU cycle of $t{d_i}$	${T^{L\_ct}}$	The finite set of the computation delay locally
$t_{t{d_i}}^{L\_ct}$	The computation delay required by $t{d_i}$ to process ${b_{t{d_i}}}$ locally	${E^{L\_ce}}$	The finite set of the computation energy consumption locally	$e_{t{d_i}}^{L\_ce}$	The computation energy consumption required by $t{d_i}$ to process ${b_{t{d_i}}}$ locally.
$par{a^S}$	The finite set of full offloading parameters	${f^S}$	The computational capability of the edge server	${\chi ^S}$	The energy coefficient
${P^{S\_ct}}$	The finite set of computation delay pricing	$p_{t{d_i}}^{S\_ct}$	The computation delay pricing of $t{d_i}$	$T{P^S}$	The finite set of transmission power
$tp_{t{d_i}}^S$	The transmission power between $t{d_i}$ and the edge server	$T{R^S}$	The finite set of the transmission rate	$tr_{t{d_i}}^S$	Transmission rate between $t{d_i}$ and the edge server,
${T^S}$	The finite set of the total delay on the edge server	${\alpha _{t{d_i}}}$, $\; {\beta _{t{d_i}}}$	The risk attitude coefficient	$t_{t{d_i}}^S$	The total delay required by $t{d_i}$ to process ${b_{t{d_i}}}$ on the edge server
${T^{S\_ct}}$	The finite set of the computation delay on the edge server	$t_{t{d_i}}^{S\_ct}$	The computation delay required by $t{d_i}$ to process ${b_{t{d_i}}}$ on the edge server	$T_{t{d_i}}^{S\_tt}$	The finite set of the transmission delay on the edge server
$t_{t{d_i}}^{S\_tt}$	The transmission delay required by $t{d_i}$ to process ${b_{t{d_i}}}$ on the edge server	${E^S}$	The finite set of the total energy consumption on the edge server	$e_{t{d_i}}^S$	The total energy consumption required by $t{d_i}$ to process ${b_{t{d_i}}}$on the edge server.
${E^{S\_ce}}$	The finite set of the computation energy consumption on the edge server.	$e_{t{d_i}}^{S\_ce}$	The computation energy consumption required by $t{d_i}$ to process ${b_{t{d_i}}}$ on the edge server	${E^{S\_te}}$	The finite set of the transmission energy consumption on the edge server
$e_{t{d_i}}^{S\_te}$	The transmission energy consumption required by $t{d_i}$ to process ${b_{t{d_i}}}$ on the edge server	${C^{S\_ct}}$	The finite set of the payment	$T^{S\_ct}$	The finite set of the computation delay on the edge server
$c_{t{d_i}}^{S\_ct}$	The computation delay cost required by $t{d_i}$ to process ${b_{t{d_i}}}$ on the edge server	$par{a^{PO}}$	The finite set of partial offloading parameters	${T^{PO}}$	The finite set of the total delay in partial offloading
$t_{t{d_i}}^{PO}$	Denotes the delay required by $t{d_i}$ to process ${b_{t{d_i}}}$ in partial offloading,	${E^{PO}}$	The finite set of the total energy consumption in partial offloading	$e_{t{d_i}}^{PO}$	The energy consumption required by $t{d_i}$ to process ${b_{t{d_i}}}$ in partial offloading
$c^{PO}$	The finite set of the total delay in partial offloading	${C^{PO\_ct}}$	The computation delay cost required by $t{d_i}$ to process ${b_{t{d_i}}}$ in partial offloading		

### System model description

In the concerned scenario, we consider a medical image cloud that includes an edge server and multiple terminal devices. The edge server provides storage and computing services for users, solving the problem of limited resources for terminal devices. The terminal devices are used by doctors, which include desktops, laptops, tablets and super beans. Each terminal device is equipped with computing resources for processing diagnosis tasks.

As the computing resources of terminal devices are limited, they cannot meet the needs of massive medical image diagnosis tasks. Therefore, users will offload part or all diagnosis tasks to the edge server. Such behavior of users carries a risk-aversion or risk-seeking attitude. The edge server typically charges a payment to share their resources. In addition, terminal devices and the edge server cause delay and energy consumption when performing diagnosis tasks. To clearly explain the offloading process of the image diagnosis task in the medical image cloud, it is formalized as follows:

***Definition* 1**. Computation offloading system model.

The computation offloading system model $C{O^{SH}} = \left( {TD,B,\mu ,\phi ,\xi ,par{a^L},par{a^S},par{a^{PO}}} \right)$ is an eight-tuple, the details of which are shown in the Supplemental Information.

### Offloading modes

**Figure 1:**
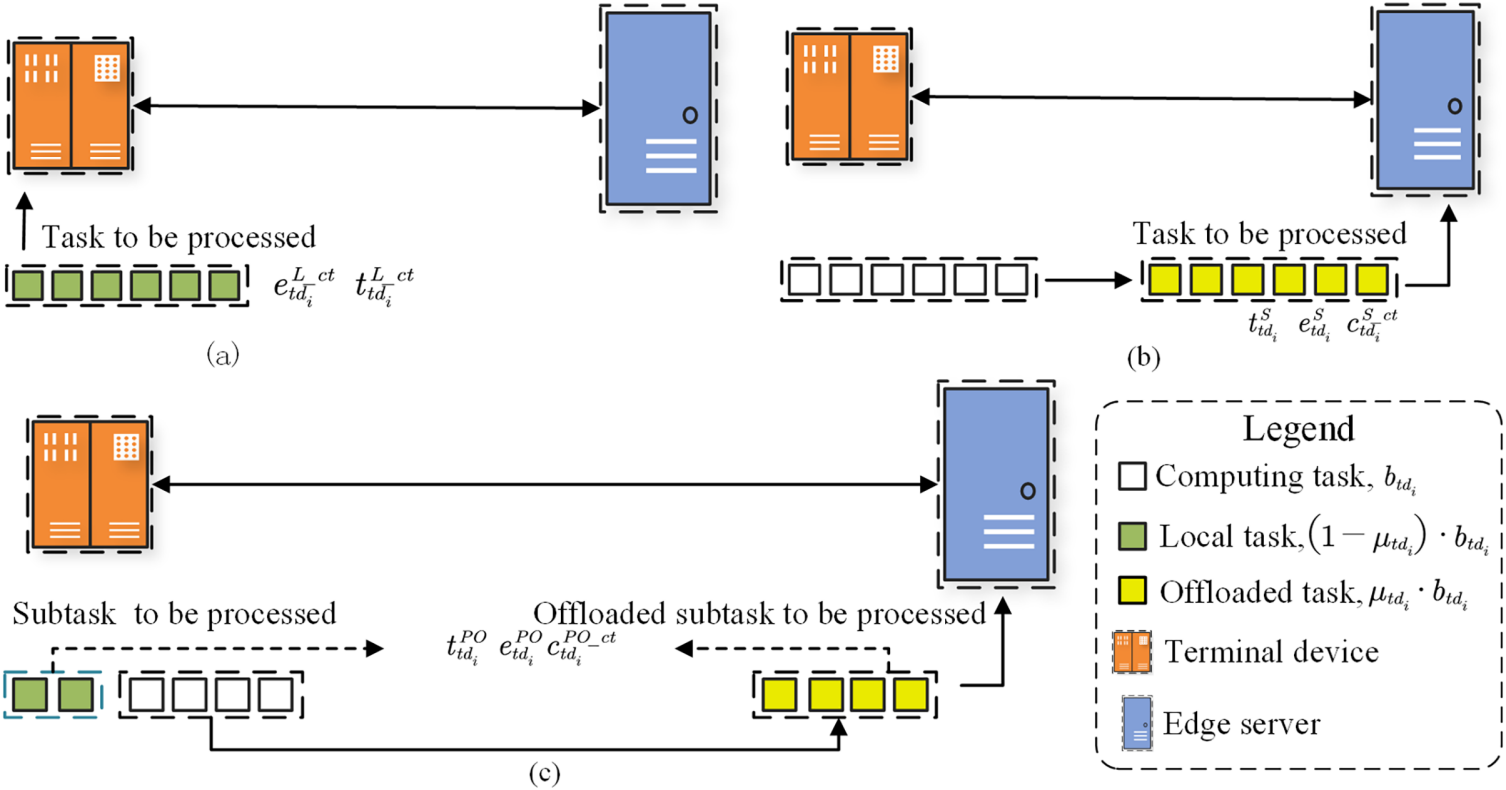
Three offloading modes for diagnosis tasks: local computing, full offloading and partial offloading.

Each terminal device has one or more medical image diagnosis tasks to perform. As shown in Fig. 1, there are three offloading modes for the diagnosis tasks: local computing, full offloading and partial offloading. Each offloading mode can be conceived as three-stages, including the sending, processing and feedback steps. First, the part or full diagnosis tasks are sent from $t{d_i}$ to the edge server. Second, the diagnosis task offloaded is processed on the edge server. Third, the processed results are feedback to $\; t{d_i}$. Next, the computing methods of three objectives in different modes are as follows.

#### Local computing

In the local computing mode, as illustrated in Fig. 1, users execute the diagnosis task ${b_{t{d_i}}}$ [bits] only using the computing resources of terminal devices, where the offloading proportion ${\mu _{t{d_i}}} = 0$. For terminal device $t{d_i}$, the local computation delay $t_{t{d_i}}^{L\_ct}$ [s] of processing ${b_{t{d_i}}}$ can be given by

(1) $$t_{t{d_i}}^{L\_ct} = \left( {{b_{t{d_i}}}\cdot \phi } \right)/f_{t{d_i}}^L$$

Besides the required computation delay, each diagnosis task also consumes some computation energy. Therefore, the local computation energy consumption $e_{t{d_i}}^{L\_ce}$ [J] required by $t{d_i}$ to process ${b_{t{d_i}}}$ can be given by

(2) $$e_{t{d_i}}^{L\_ce} = \chi _{t{d_i}}^L\cdot {b_{t{d_i}}}\cdot \phi$$

#### Full computing

**Figure 2:**
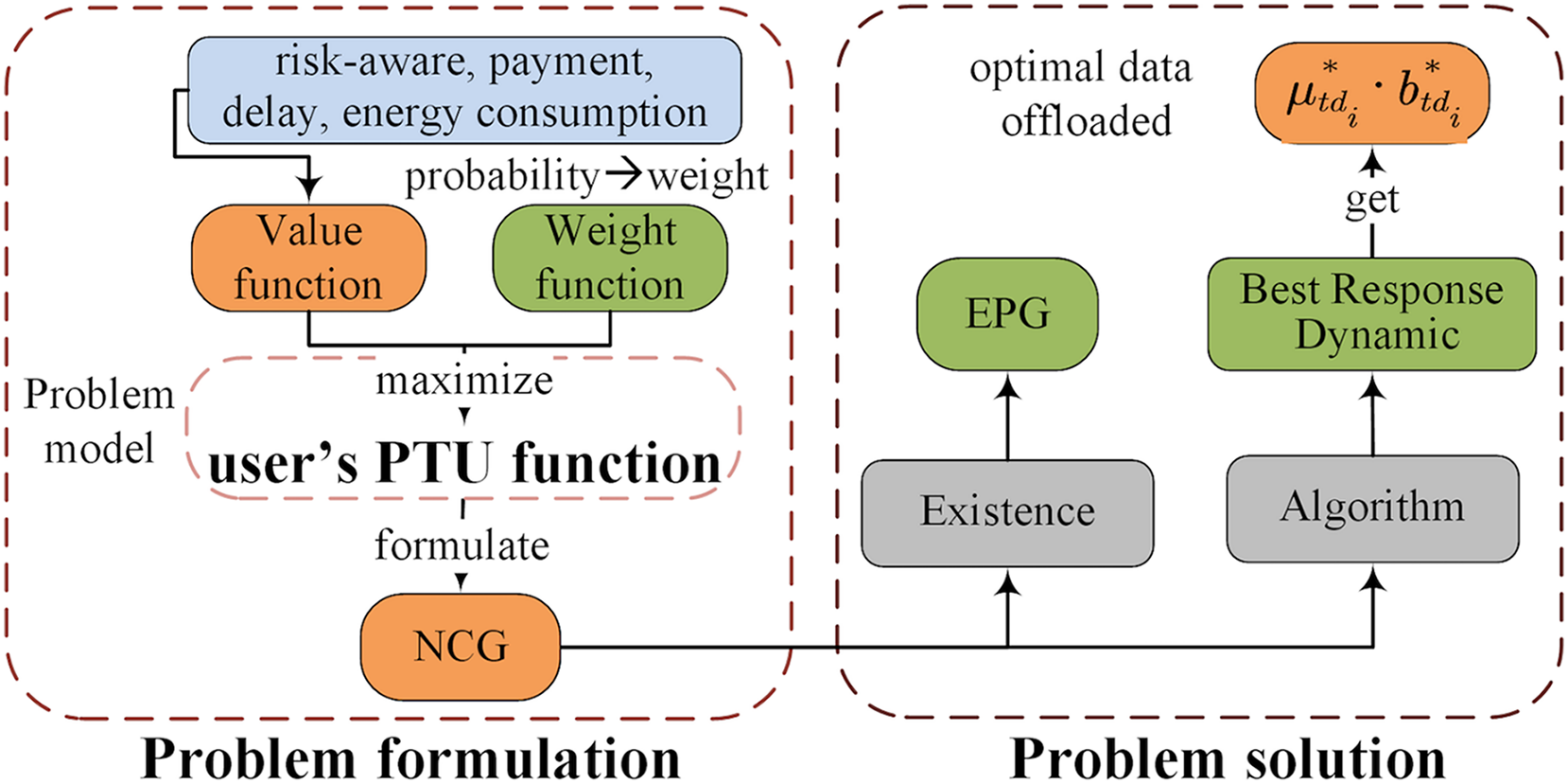
The framework of the multi-user multi-objective computation offloading method for medical image diagnosis task.

In the full offloading mode, as illustrated in Fig. 2, the diagnosis task had to be performed completely on the edge server, where the offloading proportion ${\mu _{t{d_i}}} = 1$. Therefore, the transmission delay $t_{t{d_i}}^{S\_tt}$ [s] required by $t{d_i}$ to process ${b_{t{d_i}}}$ on the edge server *via* the uplink channel can be given by

(3) $$t_{t{d_i}}^{S\_tt} = {b_{t{d_i}}}/tr_{t{d_i}}^S$$

The transmission energy consumption $e_{t{d_i}}^{S\_te}$ [J] required by $t{d_i}$ to process ${b_{t{d_i}}}$ on the edge server can be given by

(4) $$e_{t{d_i}}^{S\_te} = tp_{t{d_i}}^S\cdot t_{t{d_i}}^{S\_tt} = \left( {tp_{t{d_i}}^S\cdot {b_{t{d_i}}}} \right)/tr_{t{d_i}}^S$$

Next, the edge server will use some of the computing resources to perform ${b_{t{d_i}}}$. Therefore, the computation delay $t_{t{d_i}}^{S\_ct}$ [s] required by $t{d_i}$ to process ${b_{t{d_i}}}$ on the edge server can be given by

(5) $$t_{t{d_i}}^{S\_ct} = \left( {{b_{t{d_i}}}\cdot \phi } \right)/{f^S}$$

Meanwhile, computation energy consumption is also generated. Therefore, the computation energy consumption $e_{t{d_i}}^{S\_ce}$ [J] required by $t{d_i}$ to process ${b_{t{d_i}}}$ on the edge server can be given by

(6) $$e_{t{d_i}}^{S\_ce} = {\chi ^S}\cdot {b_{t{d_i}}}\cdot \phi$$

After the diagnosis task is completed, the results will be sent back to terminal devices *via* the downlink channel. However, resembling many studies (Xian, Lu & Li, 2007; Wang et al., 2017; Cui et al., 2017; Rudenko et al., 1998), we ignore the downlink transmission delay because the results are insufficient compared to the original image data.

In summary, the total delay $t_{t{d_i}}^S$ [s] required by $t{d_i}$ to process ${b_{t{d_i}}}$ on the edge server can be given by

(7) $$t_{t{d_i}}^S = t_{t{d_i}}^{S\_ct} + t_{t{d_i}}^{S\_tt}$$

The total energy consumption $e_{t{d_i}}^S$ [J] required by $t{d_i}$ to process ${b_{t{d_i}}}$ on the edge server can be given by

(8) $$e_{t{d_i}}^S = e_{t{d_i}}^{S\_ce} + e_{t{d_i}}^{S\_te}$$

We assume that the user has to pay a fee for the edge server based on the computation delay pricing $p_{t{d_i}}^{S\_ct}$ [$/s] (see “Prospect Theoretic Utility”) and the computation delay $t_{t{d_i}}^{S\_ct}$. Therefore, the payment $c_{t{d_i}}^{S\_ct}$ [$] required by $t{d_i}$ to process ${b_{t{d_i}}}$ on the edge server can be given by

(9) $$c_{t{d_i}}^{S\_ct} = p_{t{d_i}}^{S\_ct}\cdot t_{t{d_i}}^{S\_ct} = \left( {p_{t{d_i}}^{S\_ct}\cdot {b_{t{d_i}}}\cdot \phi } \right)\!/\!{f^S}$$

To simplify the model, we assume that the configuration and transmission setting are the same for each terminal device in this article (*i.e*., $\forall {\rm \; }i{\rm \; }{\rm \epsilon }{\rm \; }1,2, \ldots ,n,$ $f_{t{d_i}}^l = f_{td}^l,$ $\chi _{t{d_i}}^l = \chi _{td}^l,$ $tp_{t{d_i}}^S = tp_{td}^S,$ and $tr_{t{d_i}}^S = tr_{td}^S$).

#### Partial computing

**Figure 3:**
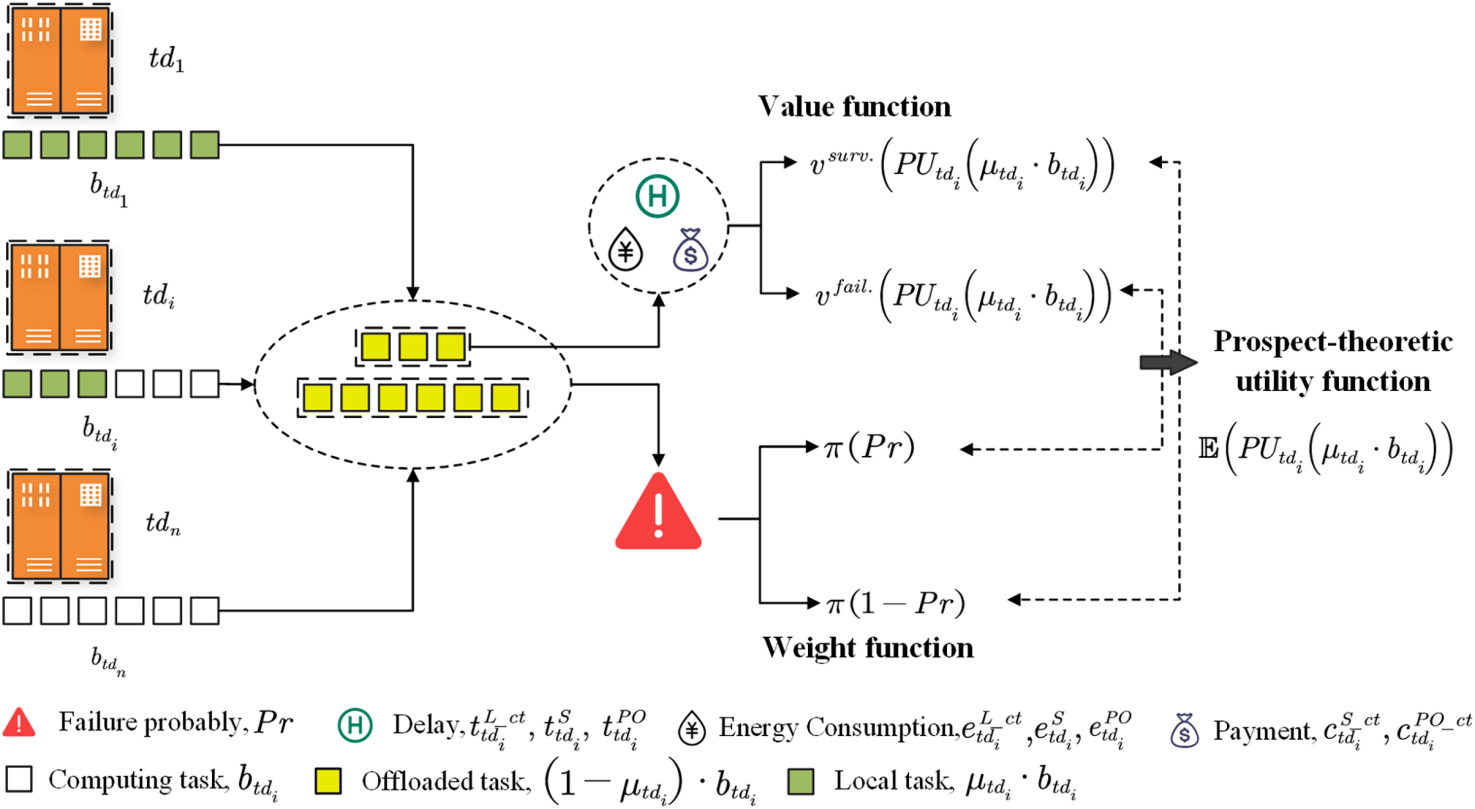
The user’s prospect theoretic utility.

In the partial offloading mode, as illustrated in Fig. 3, the diagnosis task is divided into two subtasks, where the offloading proportion ${\mu _{t{d_i}}}\in \left( {0,1} \right)$. Subtask ${\mu _{t{d_i}}}\cdot {b_{t{d_i}}}$ performed on the edge server while subtask $(1 - {\mu _{t{d_i}}})\cdot {b_{t{d_i}}}$ will be executed on $t{d_i}$. Therefore, the total delay $t_{t{d_i}}^{PO}$, total energy consumption $e_{t{d_i}}^{PO}$ and payment $c_{t{d_i}}^{PO\_ct}$ required by $t{d_i}$ to process ${b_{t{d_i}}}\;$in partial offloading can be given by

(10) $$\matrix{ {t_{t{d_i}}^{PO} = max \left\{ {(1 - {\mu _{t{d_i}}}) \cdot t_{t{d_i}}^{L\_ct},{\mu _{t{d_i}}} \cdot t_{t{d_i}}^S} \right\}} \cr {e_{t{d_i}}^{PO} = (1 - {\mu _{t{d_i}}}) \cdot e_{t{d_i}}^{L\_ce} + {\mu _{t{d_i}}} \cdot e_{t{d_i}}^S} \cr {c_{t{d_i}}^{PO\_ct} = {\mu _{t{d_i}}} \cdot c_{t{d_i}}^{S\_ct}} \cr }$$

It can be clearly seen that when the computation delay pricing $p_{t{d_i}}^{S\_ct}$ is constant, the greater amount of subtask ${\mu _{t{d_i}}}\cdot {b_{t{d_i}}}$ offload, the more payment users will pay.

## Multi-user multi-objective computation offloading for medical image diagnosis

In this section, we propose a multi-user multi-objective computation offloading for medical image diagnosis. First, the proposed framework of this system model is presented in detail. We then construct the user’s prospect theoretic utility function, formulate the offloading problem as a non-cooperative game among users, and design an algorithm to solve the problem.

### Proposed framework overview

In the above computation offloading system model, users are not always risk-neutral when deciding where to process diagnosis tasks. The reason is that the different offloading modes will yield different profits for the users. Faced with possible future gains and losses, users can hardly be in a completely neutral attitude, but will indicate different risk attitudes depending on situation.

Therefore, considering the delay, energy consumption, payment and user’s risk awareness behavioral characteristics required to complete the diagnosis tasks, we propose a multi-user multi-objective computation offloading for medical image diagnosis. The proposed framework consists of two parts, as shown in Fig. 2. (1) Problem formulation: computation offloading problem involving delay, energy consumption, payment and risk awareness behavioral characteristics, whose optimization goal is to maximize the user’s utility based on Prospect Theory. This problem is regarded as a DO problem and then formulated as an NCG among users. (2) Problem solution: the complete proof of the existence of the NE is provided by the exact potential game (EPG). Then, we propose a low-complexity computation offloading algorithm based on best response dynamics (BRD-CO), to determine the optimal data offloaded $\mu _{t{d_i}}^*\cdot b_{t{d_i}}^*$ in a distributed manner for each user.

### Problem formulation

Computation offloading problem involving risk awareness and multi-objective is regarded as a distributed optimization problem. Its optimization goal is to maximize the user’s prospect theoretic utility. Then, it is formulated as a non-cooperative game among users and solved by exact potential game.

#### Prospect theoretic utility

When users make an offloading strategy, we will analyze their risk-aware behavior using the prospect theory (PT). The prospect theory was first proposed after revising the expected utility theory based on absorbing “Allais Paradox” in 1979 (Kahneman & Tversky, 1988). The theory combines psychology and behavioral science, explicitly states that humans exhibit “loss aversion” when deciding. When faced with gains, users exhibit an attitude of risk-aversion. When faced with losses, users exhibit a risk-seeking attitude and have the principle of being more sensitive to losses than gains (Wu & Gonzalez, 1996).

Specifically, prospect theory simplifies the results by establishing appropriate reference points and a preliminary analysis of various outcomes during the editing phase. Then, the decision with the highest PTU is selected in the evaluation phase by figuring the results of the previous phase through a value function (VF) and a weight function (WF).

Each user offloads part or all of the diagnosis task to the edge server, as shown in Fig. 3. We then calculate the value of the edge server in different states based on the VF. When all users have offloaded, we calculate the failure probability of the edge server. Then, the probability is modified to a weight according to the WF. Finally, we obtain the user’s PTU by multiplying the corresponding value and weight. The specific definitions of VF and WF are as follows.

##### Value function

The value function mainly reflects the subjective value of users, following the principle of PT (Vamvakas, Tsiropoulou & Papavassiliou, 2019b), which can be given by

(11) $$v\left( {P{U_{t{d_i}}}} \right) = {\rm{ }}\left\{ {\matrix{
   {{{(P{U_{t{d_i}}} - P{U_0})}^{{\alpha _{t{d_i}}}}},} & {if{\rm{ }}P{U_{t{d_i}}} - P{U_0} \ge 0}  \cr 
   { - {k_{t{d_i}}} \cdot {{(P{U_0} - P{U_{t{d_i}}})}^{{\beta _{t{d_i}}}}},} & {if{\rm{ }}P{U_{t{d_i}}} - P{U_0} < 0}  \cr 

 } } \right.$$

Inspired by Tram, Tham & Niyato (2014), Zhou, Tham & Motani (2017), Li et al. (2019a), $P{U_0} = \xi \cdot {\rm log}\left( {1 + {b_{t{d_i}}}} \right) \cdot {\lambda _3}$ denotes the reference point, expressing the user’s anticipated profit by fully processing diagnosis task ${b_{t{d_i}}}$ at $\; t{d_i}$. $P{U_{t{d_i}}}$ represents the user’s actual profit after offloading part or all of the diagnosis task ${b_{t{d_i}}}$ to the edge server, and is given by (12) below. ${\alpha _{t{d_i}}}$ and ${\beta _{t{d_i}}}$ represent risk attitude coefficient, which are $0 \le {\alpha _{t{d_i}}},{\beta _{t{d_i}}} \le 1$. As the ${\alpha _{t{d_i}}}$ and ${\beta _{t{d_i}}}$ increase, the risk taken by the user becomes greater. ${\alpha _{t{d_i}}} = {\beta _{t{d_i}}} = 1$, the user is risk-neutral. ${k_{t{d_i}}}$ is the loss aversion coefficient. ${k_{t{d_i}}} > 1$ indicates that users are more stimulated by losses instead of than gains. Moreover, the user can adjust ${\alpha _{t{d_i}}}$, $\; {\beta _{t{d_i}}}$ and ${k_{t{d_i}}}$ in different environments. For simplicity, we attempt to assume ${\alpha _{t{d_i}}} = {\beta _{t{d_i}}}\;$in this article.

${\lambda _1}$, ${\lambda _2}$ and ${\lambda _3}$ denote the multi-objective weight coefficient, *i.e*., delay weight, energy consumption weight and payment weight. We map these different measures into the same dimension, where $0 \le {\lambda _1}$, ${\lambda _2}$, ${\lambda _3} \le 1$. For delay-sensitive tasks, ${\lambda _1}\;$is larger than ${\lambda _2}$ and ${\lambda _3}$. For energy-sensitive tasks and payment-sensitive tasks, ${\lambda _2}\;$and ${\lambda _3}$ are relatively large.

(12) $$\eqalign{
  & P{U_{t{d_i}}}\left( {{\mu _{t{d_i}}} \cdot {b_{t{d_i}}}} \right) =   \cr 
  & \left\{ {\matrix{
   & {\xi  \cdot \log \left( {1 + {b_{t{d_i}}}} \right) \cdot {\lambda _3},}  &  {if{\mu _{t{d_i}}} = 0}  \cr 
   & {\left( {t_{t{d_i}}^{L\_ct} - t_{t{d_i}}^{PO}} \right) \cdot {\lambda _1} + \left( {e_{t{d_i}}^{L\_ct} - t_{t{d_i}}^{PO}} \right) \cdot {\lambda _2} + \left( {\xi  \cdot \log \left( {1 + {b_{t{d_i}}}} \right) - c_{t{d_i}}^{PO\_ct}} \right) \cdot {\lambda _3},}  & 
   {if{\mu _{t{d_i}}} \ne 0{\rm{\,}}and{\rm{\,}}edge{\rm{\,}}server{\rm{\,}}survives}  \cr 
    & {\left( {e_{t{d_i}}^{L\_ct} - t_{t{d_i}}^{PO}} \right) \cdot {\lambda _2} + \left( {\left( {1 - {\mu _{t{d_i}}}} \right) \cdot \xi  \cdot \log \left( {1 + {b_{t{d_i}}}} \right) - c_{t{d_i}}^{PO\_ct}} \right) \cdot {\lambda _3},}  &  
   {if{\mu _{t{d_i}}} \ne 0{{\ }}and{{\ }}edge{{\ }}server{{\ }}fails}  \cr 

 } } \right.} $$

Given the weak computing capacity of a terminal device, it cannot meet the computing needs of a massive medical image. As a common resource, the edge server can provide services for all users. Every user can enjoy edge server services, but the computing resources of the edge server are limited. There will be serious negative effects when the resources use exceeds the boundary. Here, we mainly divide it into two situations.

Situation 1: edge server survives. There may be some signal interference or channel congestion, resulting in reduced transmission efficiency, but the edge server remains capable of diagnosis tasks of terminal devices.

Situation 2: edge server failures. Excessive competition for computing resources on edge server, terminal devices will no longer be able to enjoy services once edge server is shutdown.

The first branch of (12) denotes the actual profit of the user performing tasks entirely on the terminal device. The second branch of (12) corresponds to situation 1, where the user’s actual profit depends primarily on the delay, energy consumption and payment after executing all the images. Quite the opposite, the third branch of (12) corresponds to situation 2, where the user’s actual benefit is determined by the energy consumption and payment. The reason is that the edge server is shutdown and is no longer able to get a delay gain after processing the image.

Therefore, the living state of the edge server directly affects the user’s actual profits. In situation 1, the value function of the user should be determined by the first branch of (11) and the second branch of (12), which is defined as

(13) $$\eqalign{
   {v^{surv}}\left( {P{U_{t{d_i}}}\left( {{\mu _{t{d_i}}} \cdot {b_{t{d_i}}}} \right)} \right) = & {(P{U_{td}}_i - P{U_0})^{{\alpha _{t{d_i}}}}}  \cr 
  &  = {\left( {\left( {t_{t{d_i}}^{{L_{ct}}} - t_{t{d_i}}^{PO}} \right) \cdot {\lambda _1} + {\mu _{t{d_i}}} \cdot \left( {\left( {e_{t{d_i}}^{{L_{ce}}} - e_{t{d_i}}^S} \right) \cdot {\lambda _2} - p_{t{d_i}}^{S\_ct} \cdot t_{t{d_i}}^{S\_ct} \cdot {\lambda _3}} \right)} \right)^{{\alpha _{t{d_i}}}}} \cr} $$

In situation 2, the value function of the user should be resolute by the second branch of (11) and the third branch of (12), which is defined as

(14) $$\eqalign{
  & {v^{fail}}(P{U_{t{d_i}}}({\mu _{t{d_i}}} \cdot {b_{t{d_i}}})) =  - {k_{t{d_i}}} \cdot {(P{U_0} - P{U_{td}}_i)^{{\alpha _{t{d_i}}}}}  \cr 
  & \quad \quad \quad \quad \quad \quad \quad \quad \quad  =  - {k_{t{d_i}}} \cdot {\mu _{t{d_i}}}^{{\alpha _{t{d_i}}}} \cdot ((\xi  \cdot \log (1 + {b_{t{d_i}}}) + p_{t{d_i}}^{S\_ct} \cdot t_{t{d_i}}^{S\_ct}) \cdot {\lambda _3}^{{\alpha _{t{d_i}}}}  \cr 
  & \quad \quad \quad \quad \quad \quad \quad \quad \quad  - (e_{t{d_i}}^{L\_ce} - e_{t{d_i}}^S) \cdot {\lambda _2}) \cr} $$

According to the math characteristics of the value function, (13) must be a positive constant, and (14) must be a negative constant. Thus, we can determine the boundaries of the computing delay pricing $p_{t{d_i}}^{S\_ct}$ imposed by the edge server on the user, which can be given by

(15) $$\matrix{ {\left\{ {\matrix{ {p_{t{d_i}}^{S\_ct} \gt 0} \hfill \cr { \left( {t_{t{d_i}}^L - t_{t{d_i}}^{PO}} \right)\cdot {\lambda _1} + {\mu _{t{d_i}}} \cdot \left( {\left( {e_{t{d_i}}^{L\_ce} - e_{t{d_i}}^{S}} \right) \cdot {\lambda _2} - p_{t{d_i}}^{S\_ct} \cdot t_{t{d_i}}^{S\_ct} \cdot \lambda_{3}} \right) \gt 0} \cr {\left( {\xi \cdot \log \left( {1 + {b_{t{d_i}}}} \right) + p_{t{d_i}}^{S\_ct} \cdot t_{t{d_i}}^{S\_ct}} \right)\cdot \lambda_{3} - \left( {e_{t{d_i}}^{L\_ce} - e_{t{d_i}}^{S}} \right) \cdot {\lambda _2} \gt 0} \cr } } \right.} \cr { \Rightarrow 0 \lt p_{t{d_i}}^{S\_ct} \lt \displaystyle{{ \left( {t_{t{d_i}}^{L\_ct} - t_{t{d_i}}^{PO}} \right) \cdot{\lambda _1} + \left( {e_{t{d_i}}^{L\_ce} - e_{t{d_i}}^{S}} \right) \cdot {\lambda _2}} \over {{\lambda _3} \cdot t_{t{d_i}}^{S\_ct}}}} \cr }$$

For simplicity, $\; \varphi = \displaystyle{{{\lambda _1} \cdot \left( {t_{t{d_i}}^{L\_ct} - t_{t{d_i}}^{PO}} \right) + \left( {e_{t{d_i}}^{L\_ct} - t_{t{d_i}}^{PO}} \right) \cdot {\lambda _2}} \over {{\lambda _3} \cdot t_{t{d_i}}^{S\_ct}}}$, so $p_{t{d_i}}^{S\_ct} = \omega \cdot \varphi$, where payment factor $\omega \in \left( {0,1} \right)$.

##### Weight function

The weight function reflects the degree of perception of probability. Users have different risk behaviors towards different failure probabilities of the edge server during the diagnosis task offloading (*i.e*., Gains and losses). The failure probability of the edge server is directly related to the size of the processed data. The reason is that the larger the offloaded amount, the higher the computing demand for the terminal devices. This will lead to a greater failure probability of the edge server. Inspire by Mitsis, Tsiropoulou & Papavassiliou (2020), the failure probability of the edge server $Pr$ can be given by

(16) $$Pr\left( {{\mu _{t{d_i}}}\cdot {b_{t{d_i}}}} \right) = {\left( { - 1 + \displaystyle{2 \over {1 + {e^{ - \varsigma \mathop \sum \nolimits_{i = 1}^n \phi \cdot {\mu _{t{d_i}}}\cdot {b_{t{d_i}}}}}}}} \right)^2}$$

where ${\mu _{t{d_i}}}\cdot {b_{t{d_i}}}$ represents the offload image data of the user on the edge server. $\varsigma > 0$ is a positive constant calibrating the sigmoidal curve based on the computing capabilities of the edge server (Mitsis, Tsiropoulou & Papavassiliou, 2020). The failure probability of the edge server, $0 \le Pr \le 1$, is a continuous, strictly increasing, convex, and twice differentiable function (Mitsis, Tsiropoulou & Papavassiliou, 2020).

Following the principle of PT, we convert the probability function $P$ into the weight function $\pi \left( P \right)$, which is defined as

(17) $$\pi \left( P \right) = \left\{ {\matrix{ {\displaystyle{{{P^r}} \over {{{\left( {{P^r} + {{\left( {1 - P} \right)}^r}} \right)}^{{\raise0.7ex\hbox{$1$} \!\mathord{\left/ {\vphantom {1 r}}\right.} \!\lower0.7ex\hbox{$r$}}}}}},\; \; \; \; \; \; \; \; \; \; \; \; \; if\; P{U_{t{d_i}}} - P{U_0} \ge 0} \cr {\displaystyle{{{P^\delta }} \over {{{\left( {{P^\delta } + {{\left( {1 - P} \right)}^\delta }} \right)}^{{\raise0.7ex\hbox{$1$} \!\mathord{\left/ {\vphantom {1 \delta }}\right.} \!\lower0.7ex\hbox{$\delta $}}}}}},\; \; \; \; \; \; \; \; \; \; \; \; if\; P{U_{t{d_i}}} - P{U_0} < 0} \cr } } \right.$$

The parameter $\gamma ,\; \delta < 1\;$denote the risk attitude of the user to gains and losses in making an offloading strategy. $\pi \left( P \right)$ is the increment function of $\; P$. When $P$ is a small probability event approaching 0, users show risk-seeking attitude (*i.e*., $\pi \left( P \right) > P$). For the events with medium probability and high probability, the users show the attitude of risk aversion (*i.e*., $P > \pi \left( P \right)$). In other words, low-probability events tend to be overestimated, and the converse holds (Monderer & Shapley, 1996).

Combining (11)–(17), following the principle of PT, the user’s prospect theoretic utility function comprises the value function and the weight function, which is defined as follows

(18) $$\eqalign{ {\rm {\mathbb E}}\left( {P{U_{t{d_i}}}\left( {{\mu _{t{d_i}}}\cdot {b_{t{d_i}}}} \right)} \right) =\ &  {v^{surv}}\left( {P{U_{t{d_i}}}\left( {{\mu _{t{d_i}}}\cdot {b_{t{d_i}}}} \right)} \right)\cdot {\pi ^{surv}}\left( {1 - Pr} \right) \cr& + {v^{fail}}\left( {P{U_{t{d_i}}}\left( {{\mu _{t{d_i}}}\cdot {b_{t{d_i}}}} \right)} \right)\cdot {\pi ^{fail}}\left( {Pr} \right) } $$

where ${\pi ^{surv}}\left( {1 - Pr} \right)$ denotes the weight of the edge server survives (*i.e*., Gains). ${\pi ^{fail}}\left( {Pr} \right)$ represents the weight of the edge server fails (*i.e*., Losses). The definition is as follows

(19) $${\pi ^{surv}}\left( {1 - Pr} \right) = \displaystyle{{{P^r}} \over {{{\left( {{P^r} + {{\left( {1 - P} \right)}^r}} \right)}^{{\raise0.7ex\hbox{$1$} \!\mathord{\left/ {\vphantom {1 r}}\right.} \!\lower0.7ex\hbox{$r$}}}}}},\; \; \; \; \; \; \; \; \; \; \; \; \; if\; P{U_{t{d_i}}} - P{U_0} \ge 0$$

(20) $${\pi ^{fail}}\left( {Pr} \right) = \displaystyle{{{P^\delta }} \over {{{\left( {{P^\delta } + {{\left( {1 - P} \right)}^\delta }} \right)}^{{\raise0.7ex\hbox{$1$} \!\mathord{\left/ {\vphantom {1 \delta }}\right.} \!\lower0.7ex\hbox{$\delta $}}}}}},\; \; \; \; \; \; \; \; \; \; \; \; if\; P{U_{t{d_i}}} - P{U_0} < 0$$

#### Problem model

To maximize the prospect theoretic utility of each user, the computation offloading problem for medical image diagnosis task involving risk awareness and multi-objective (*i.e*., delay, energy consumption and payment) is, therefore, represented as a distributed optimization problem as follows

(21) $${\rm max}\; {\rm {\mathbb E}}\left( {P{U_{t{d_i}}}\left( {{\mu _{t{d_i}}}\cdot {b_{t{d_i}}},{\boldsymbol{\mu }_{ - \boldsymbol{t}{\boldsymbol{d}_{\boldsymbol{i}}}}}\cdot {\boldsymbol{b}_{ - \boldsymbol{t}{\boldsymbol{d}_{\boldsymbol{i}}}}}} \right)} \right)\; \; \; \; \; \; s.t.\; \; 0 \le {\mu _{t{d_i}}} \le 1$$

where ${\boldsymbol{\mu }_{ - \boldsymbol{t}{\boldsymbol{d}_{\boldsymbol{i}}}}}\cdot {\boldsymbol{b}_{ - \boldsymbol{t}{\boldsymbol{d}_{\boldsymbol{i}}}}}$ is the amount of image data offloaded by the rest of the terminal devices except for the terminal device $t{d_i}$. The above problem is defined as a non-cooperative game among users as follows

(22) $${G_{dop}} = \left[ {TD,O{S_{t{d_i}}},{\rm {\mathbb E}}\left( {P{U_{t{d_i}}}\left( {{\mu _{t{d_i}}}\cdot {b_{t{d_i}}},{\boldsymbol{\mu }_{ - \boldsymbol{t}{\boldsymbol{d}_{\boldsymbol{i}}}}}\cdot {\boldsymbol{b}_{ - \boldsymbol{t}{\boldsymbol{d}_{\boldsymbol{i}}}}}} \right)} \right)} \right]$$

where $TD$ is the finite set of the user terminal devices, $O{S_{t{d_i}}}$ is the offloading strategies space of $t{d_i}$, and ${\rm {\mathbb E}}\left( {P{U_{t{d_i}}}\left( {{\mu _{t{d_i}}}\cdot {b_{t{d_i}}},{\boldsymbol{\mu }_{ - \boldsymbol{t}{\boldsymbol{d}_{\boldsymbol{i}}}}}\cdot {\boldsymbol{b}_{ - \boldsymbol{t}{\boldsymbol{d}_{\boldsymbol{i}}}}}} \right)} \right)$ reflects the prospect theoretic utility of the user *i*. The solution of ${G_{dop}}$ for the user’s optimal computation offloading strategy $\mu _{t{d_i}}^*\cdot b_{t{d_i}}^*$, the meaning of which is that PTU is greatest when the amount of data offloaded is $\mu _{t{d_i}}^*\cdot b_{t{d_i}}^*$.

***Definition* 2**. Nash equilibrium.

An image data offloading vector $\boldsymbol{\mu }_{\boldsymbol{t}{\boldsymbol{d}_{\boldsymbol{i}}}}^{\boldsymbol{*}}\cdot \boldsymbol{b}_{\boldsymbol{t}{\boldsymbol{d}_{\boldsymbol{i}}}}^{\boldsymbol{*}} = \left\{ {\mu _{t{d_1}}^*\cdot b_{t{d_1}}^*,\mu _{t{d_2}}^*\cdot b_{t{d_2}}^*, \ldots ,\mu _{t{d_n}}^*\cdot b_{t{d_n}}^*} \right\}$ in the strategy space $\mu _{t{d_i}}^*\cdot b_{t{d_i}}^*\in O{S_{t{d_i}}} = \left[ {0,{b_{t{d_i}}}} \right]$ is a Nash Equilibrium point if for every user *i* the following condition holds true

(23) $$\eqalign{& {\rm {\mathbb E}}\left( {P{U_{t{d_i}}}\left( {\mu _{t{d_i}}^*\cdot b_{t{d_i}}^*,\boldsymbol{\mu }_{ - \boldsymbol{t}{\boldsymbol{d}_{\boldsymbol{i}}}}^{\boldsymbol{*}}\cdot \boldsymbol{b}_{ - \boldsymbol{t}{\boldsymbol{d}_{\boldsymbol{i}}}}^{\boldsymbol{*}}} \right)} \right) \gt {\rm {\mathbb E}}\left( {P{U_{t{d_i}}}\left( {{\mu _{t{d_i}}}\cdot {b_{t{d_i}}},\boldsymbol{\mu }_{ - \boldsymbol{t}{\boldsymbol{d}_{\boldsymbol{i}}}}^{\boldsymbol{*}}\cdot \boldsymbol{b}_{ - \boldsymbol{t}{\boldsymbol{d}_{\boldsymbol{i}}}}^{\boldsymbol{*}}} \right)} \right) \cr&  {\rm for \; all}\;{\mu _{t{d_i}}}\cdot {b_{t{d_i}}}\in O{S_{t{d_i}}} } $$

The meaning of the Nash equilibrium point is that, no player (users in our problem) can further increase the cost (user’s prospect theoretic utility in our problem) by unilaterally changing his strategy while the other player’s strategy (computation offloading strategy in our problem) remains unchanged.

### Problem solution

In this section, we first prove the existence of NE points for the NCG by EPG. Then, a computation offloading algorithm based on best response dynamics is proposed to solve the problem. Finally, we discuss the time complexity of the proposed algorithm.

#### The existence of NE point

To prove ${G_{dop}}$ has at least one NE point, which means as a solution to maximize the distributed optimization problem, the exact potential game is adopted. The main reason for this design is that not all NCGs have an NE point and can reach algorithmic convergence. An exact potential game with a limited set of strategies converges to at least one NE point, regardless of the starting point.

***Definition* 3**. Exact potential game. The ${G_{dop}} = \left[ {TD,O{S_{t{d_i}}},{\rm {\mathbb E}}\left( {P{U_{t{d_i}}}  \left( {{\mu _{t{d_i}}}\cdot {b_{t{d_i}}},  {\boldsymbol{\mu }_{ - \boldsymbol{t}{\boldsymbol{d}_{\boldsymbol{i}}}}}\cdot {\boldsymbol{b}_{ - \boldsymbol{t}{\boldsymbol{d}_{\boldsymbol{i}}}}}} \right)} \right)} \right]$ is an EPG if there is an exact potential function $\Phi \left( {{\boldsymbol{\mu }_{\boldsymbol{t}{\boldsymbol{d}_{\boldsymbol{i}}}}}\cdot {\boldsymbol{b}_{\boldsymbol{t}{\boldsymbol{d}_{\boldsymbol{i}}}}}} \right)$ that for all $t{d_i}\in TD$ satisfies the following conditions

(24) $$\eqalign{ {\Phi \left( {{{\mu }^\prime _{{\boldsymbol{t}}{{\boldsymbol{d}}_{\boldsymbol{i}}}}} \cdot {{\boldsymbol{b}}^\prime _{{\boldsymbol{t}}{{\boldsymbol{d}}_{\boldsymbol{i}}}}}} \right) - \Phi \left( {{{\mu }_{{\boldsymbol{t}}{{\boldsymbol{d}}_{\boldsymbol{i}}}}} \cdot {{\boldsymbol{b}}_{{\boldsymbol{t}}{{\boldsymbol{d}}_{\boldsymbol{i}}}}}} \right)} =\ &  {{\rm {\mathbb E}}\left( {P{U_{t{d_i}}}\left( {{\mu^\prime  _{t{d_i}}} \cdot {b^\prime _{t{d_i}}},{{\mu }_{ - {\boldsymbol{t}}{{\boldsymbol{d}}_{\boldsymbol{i}}}}} \cdot {{\boldsymbol{b}}_{ - {\boldsymbol{t}}{{\boldsymbol{d}}_{\boldsymbol{i}}}}}} \right)} \right)} \cr& - {{\rm {\mathbb E}}\left( {P{U_{t{d_i}}}\left( {{\mu _{t{d_i}}} \cdot {b_{t{d_i}}},{{\mu }_{ - {\boldsymbol{t}}{{\boldsymbol{d}}_{\boldsymbol{i}}}}} \cdot {{\boldsymbol{b}}_{ - {\boldsymbol{t}}{{\boldsymbol{d}}_{\boldsymbol{i}}}}}} \right)} \right)} \cr&  {for\;all\;{\mu _{t{d_i}}} \cdot {b_{t{d_i}}},{\mu^\prime _{t{d_i} }} \cdot {b_{t{d_i}^\prime }}{\rm \epsilon }O{S_{t{d_i}}},{{\mu }_{ - {\boldsymbol{t}}{{\boldsymbol{d}}_{\boldsymbol{i}}}}} \cdot {{\boldsymbol{b}}_{ - {\boldsymbol{t}}{{\boldsymbol{d}}_{\boldsymbol{i}}}}}{\rm \epsilon }O{S_{ - t{d_i}}}} \cr }  $$

**Theorem 1**. The ${G_{dop}} = \left[ {TD,O{S_{t{d_i}}},{\rm {\mathbb E}}\left( {P{U_{t{d_i}}}\left( {{\mu _{t{d_i}}}\cdot {b_{t{d_i}}},{\boldsymbol{\mu }_{ - \boldsymbol{t}{\boldsymbol{d}_{\boldsymbol{i}}}}}\cdot {\boldsymbol{b}_{ - \boldsymbol{t}{\boldsymbol{d}_{\boldsymbol{i}}}}}} \right)} \right)} \right]$ is an exact potential game and has at least one Nash equilibrium point $\boldsymbol{\mu }_{\boldsymbol{t}{\boldsymbol{d}_{\boldsymbol{i}}}}^{\boldsymbol{*}}\cdot \boldsymbol{b}_{\boldsymbol{t}{\boldsymbol{d}_{\boldsymbol{i}}}}^{\boldsymbol{*}} = \left\{ {\mu _{t{d_1}}^*\cdot b_{t{d_1}}^*,\mu _{t{d_2}}^*\cdot b_{t{d_2}}^*, \ldots ,\mu _{t{d_n}}^*\cdot b_{t{d_n}}^*} \right\}$. (Due to space limitation, the proof of theorem 1 is shown in the Supplemental Information).

#### Computation offloading algorithm based on best response dynamics

Given that we have already proven that the ${G_{dop}}$ belongs to the class of EPG as stated above, and exists at least one NE point. In an exact potential game, the NE point can always be reached after a finite number of iterations, which is called the finitely increasing property (Yang et al., 2020). Therefore, the best response dynamics is adopted to determine each user’s optimal computation offloading strategy $\mu _{t{d_i}}^*\cdot b_{t{d_i}}^*$ (*i.e*., converged to a NE point) in a distributed manner through a finite number of iterations, when the computation offloading strategy of other users is determined (Topkis, 1998; Milgrom & Roberts, 1990), as follows

(25) $$BR\left( {{\mu _{t{d_i}}}\cdot {b_{t{d_i}}},{\boldsymbol{\mu }_{ - \boldsymbol{t}{\boldsymbol{d}_{\boldsymbol{i}}}}}\cdot {\boldsymbol{b}_{ - \boldsymbol{t}{\boldsymbol{d}_{\boldsymbol{i}}}}}} \right) = \mu _{t{d_i}}^*\cdot b_{t{d_i}}^* = {\it arg} {\rm \; max}\;{\rm {\mathbb E}}\left( {P{U_{t{d_i}}}\left( {{\mu _{t{d_i}}}\cdot {b_{t{d_i}}},{\boldsymbol{\mu }_{ - \boldsymbol{t}{\boldsymbol{d}_{\boldsymbol{i}}}}}\cdot {\boldsymbol{b}_{ - \boldsymbol{t}{\boldsymbol{d}_{\boldsymbol{i}}}}}} \right)} \right)$$

From the above discussion, we propose a low-complexity computation offloading algorithm based on best response dynamics (BRD-CO) to determine each user’s computation offloading strategy. To more clearly describe the workflow of the BRD-CO algorithm, described briefly in Table 3, the algorithm follows a pseudo-code. The BRD-CO algorithm comprises three parts: the first part is a line 1–4, the initial definition of parameters, including the number of iterations, the user *i’s* computation offloading strategy and the convergence of the algorithm. The second part is lines 9–14, which calculates the user *i’s* prospect theoretic utility. In each iteration, first, we calculate the delay, energy consumption and payment in three offloading modes *via* (1)–(10). Second, using (13) and (14), the user *i’s* value in the survival and failure state of the edge server can be obtained. Then, we calculate the failure probability of the edge server *via* (16) and use (19) and (20) convert probability to weight. Finally, taking advantage of (18), we get the user $\; {i}^{\prime}s$ prospect theoretic utility. The third part is 16–20 lines, which determine the user ${i}^{\prime}s$ optimal offloading strategy. We calculate the user *i’s* offload strategy *via* (25) each time. If two adjacent times strategies are the same, the strategy is called the optimal offload strategy for the user *i*.

**Table 3:** BRD-CO algorithm.

Algorithm 1: BRD-CO algorithm	
**Input:** all paraments of $C{O^{SH}}$ model, ${b_{t{d_i}}}$, $\phi$, $\; {\mu _{t{d_i}}}$, $\xi$, $f_{t{d_i}}^L$, $\; \chi _{t{d_i}}^L$, $\; {f^S}$, ${\chi ^S}$, $tp_{t{d_i}}^S$, $tr_{t{d_i}}^S$, ${\lambda _1}$, $\; {\lambda _2}$, $\; {\lambda _3}$, ${\rm \; }\forall {\rm \; }i{\rm \; }{\rm \epsilon }{\rm \; }n$	
**Output:** optimal computation offloading strategy, $\mu _{t{d_i}}^*\cdot b_{t{d_i}}^*$	
1.	**// Initialization Paraments**
2.	$ite\leftarrow 0$ //iterations
3.	${({\mu _{t{d_i}}}\leftarrow {b_{t{d_i}}})^{ite\leftarrow 0}}$ //The initial amount of image data offloaded for the user *i*
4.	*convergence* $\leftarrow$ ***false*** //Whether the algorithm converges
5.	**While** *convergence* == ***false*** **do**
6.	$ite\leftarrow ite + 1$
7.	** While** $i < n$ **do**
8.	** // computer prospect theoretic utility**
9.	** **computer delay, energy consumption and payment *via* (1)–(10)
10.	** **computer ${v^{surv}}{\left( {P{U_{t{d_i}}}\left( {{\mu _{t{d_i}}}\cdot {b_{t{d_i}}}} \right)} \right)^{ite - 1}}$ *via* (13)
11.	** **computer ${v^{fail}}{\left( {P{U_{t{d_i}}}\left( {{\mu _{t{d_i}}}\cdot {b_{t{d_i}}}} \right)} \right)^{ite - 1}}$ *via* (14)
12.	** **obtain $Pr{\left( {{\mu _{t{d_i}}}\cdot {b_{t{d_i}}}} \right)^{ite - 1}}$ *via* (16)
13.	** **convert (16) into weight ${\pi ^{surv}}{\left( {1 - Pr} \right)^{ite - 1}}$ and ${\pi ^{fail}}{\left( {Pr} \right)^{ite - 1}}$ and *via* (19) and (20)
14.	** **computer ${\rm {\mathbb E}}{\left( {P{U_{t{d_i}}}\left( {{\mu _{t{d_i}}}\cdot {b_{t{d_i}}}} \right)} \right)^{ite - 1}}$ *via* (18)
15.	** // determine the optimal strategy**
16.	** **user *i* determines ${\left( {\mu _{t{d_i}}^*\cdot b_{t{d_i}}^*} \right)^{ite}}$ is based on $\; {\left( {\mu _{t{d_i}}^*\cdot b_{t{d_i}}^*} \right)^{ite - 1}}$ *via (25)*
17.	** End while**
18.	** If** ${\left( {\mu _{t{d_i}}^*\cdot b_{t{d_i}}^*} \right)^{ite}} = = {\left( {\mu _{t{d_i}}^*\cdot b_{t{d_i}}^*} \right)^{ite - 1}}$ **then**
19.	** ***convergence* $\leftarrow$ ***true***
20.	** End if**
21.	**End while**

#### Time complexity of BRD-CO algorithm

In this section, the time complexity of the proposed BRD-CO algorithms is discussed. From the above pseudo-code analysis, it can be seen that the solution process of the BRD-CO algorithm is iterative, and its time complexity is mainly determined by three factors: the number of iterations, the number of users, and the complexity of the utility function. Specifically, assume that the number of iterations required for Algorithm 1 is the complexity of (25) is, the number of users is. In each iteration, the formula (25) is calculated for all users. In addition, the difference between the current and previous offloading amounts is compared. If the difference is within the error range, the convergence state is adjusted to true; otherwise, it is adjusted to false. Therefore, the time complexity of the BRD-CO algorithm is *O*(*F* $\cdot$ *ite* $\cdot$ *n*).

## Numerical results

In this section, we introduce the simulation setting to build $C{O^{SH}}$. The parameter influence analysis then is exhibited from five aspects. Next, we discuss the convergence of the algorithm. Finally, we compare the proposed BRD-CO algorithm with four benchmarks and four heuristic methods.

### Simulation setup

To evaluate the parameter influence and convergence of the algorithm, we use PyCharm as the development tool for Python IDE. The performed simulations were executed on an Intel® Xeon (R) Silver 4114 CPU @ 2.20 GHz $\; \times$ 40 with 128 GB RAM.

The contents of the simulation are as follows: Suppose that 25 users simultaneously offload part of the annotation tasks to the edge server. The data used in the experiment came from the dynamic panoramic PET data set of Henan Provincial People’s Hospital. Each user’s prospect theoretic utility is calculated *via* (18). Using the BRD-CO algorithm to explore the optimal offloading strategy for each user to maximize their PTU. Inspired by Apostolopoulos, Tsiropoulou & Papavassiliou (2020), the main parameters are given in Table 4.

**Table 4:** The value for simulation parameters.

Parameters	Value	Parameters	Value	Parameters	Value
${b_{t{d_i}}}$	$10 \times {10^6} \pm {10^6}\;$bits	$\chi _{t{d_i}}^L$	$4 \times {10^{ - 9}} \pm {10^{ - 9}}$ J/CPU-cycles	$\phi$	1,000 CPU-cycles/bit
$\omega$	0.5	${k_{t{d_i}}}$	1.2	${\alpha _{t{d_i}}}$	0.2
$\xi$	5 $/bit	${\chi ^S}$	$4 \times {10^{ - 8}}$ J/CPU-cycles	${f^S}$	$6 \times {10^{10}}$ CPU-cycles/s
$f_{t{d_i}}^L$	$6 \times {10^7} \pm {10^7}\;$CPU-cycles/s	$tp_{t{d_i}}^S$	${10^9}\;$dbm	$tr_{t{d_i}}^S$	0.1 bits/s
${\lambda _1}$	1	${\lambda _2}$	0.001	${\lambda _3}$	0.1
$\gamma$	0.61	$\delta$	0.69		

### Parameter influence analysis

In “Impact of Computing Delay Pricing”, we consider the number of users (denoted by ‘N’) is set to 25 and parameter values as indicated in Table 4. However, in the rest of the analysis, a wide range of computing delay pricing $p_{t{d_i}}^{S\_ct}$, number of users, multi-objective weight coefficients ( ${\lambda _1}$, ${\lambda _2}$ and ${\lambda _3}$) and prospect theoretic parameters (risk attitude ${\alpha _{t{d_i}}}$, gain attitude $\gamma$, loss aversion ${k_{t{d_i}}}$ and loss attitude $\delta$) are considered.

#### Impact of computing delay pricing

**Figure 4:**
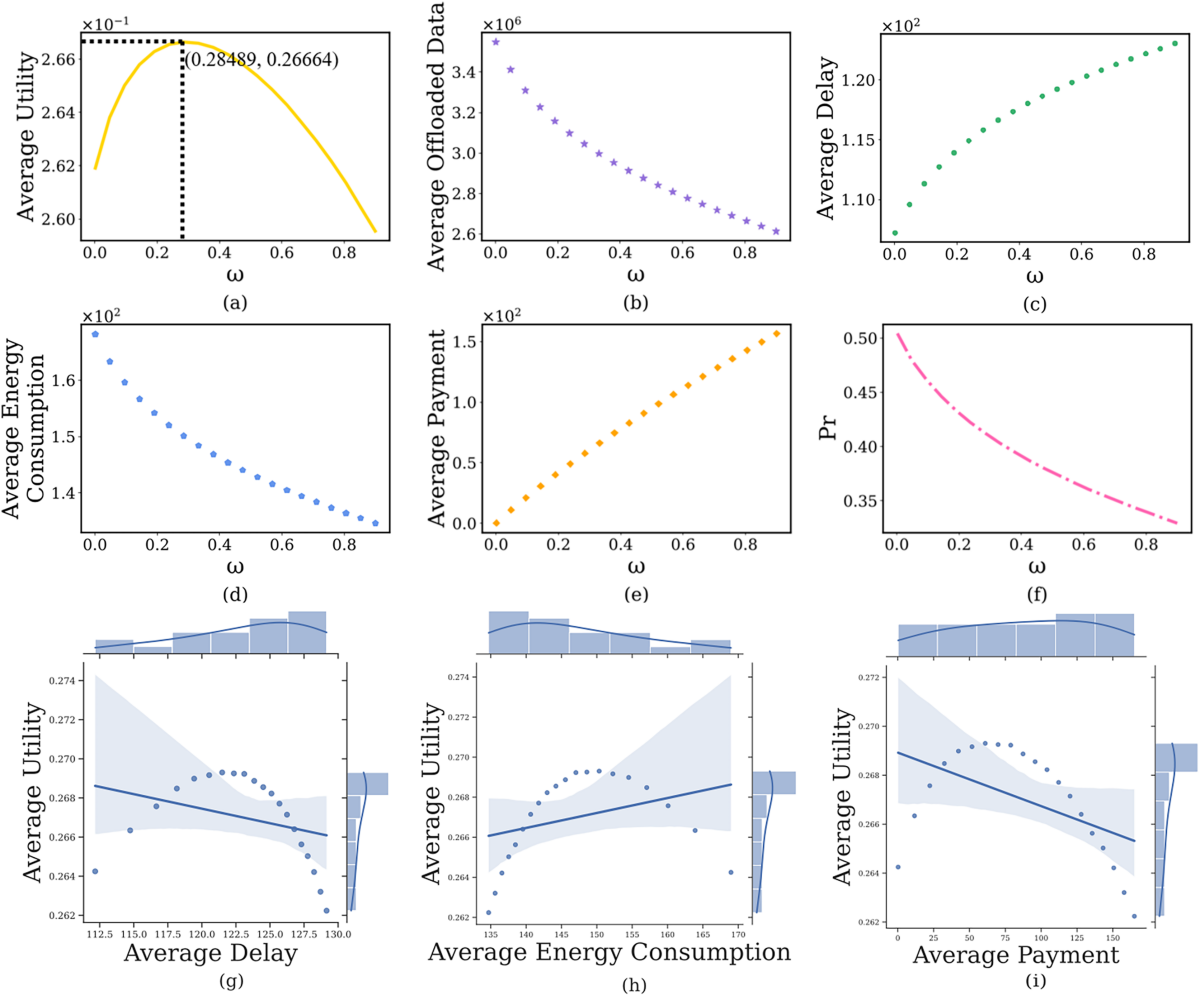
The relationship between computing delay pricing and user’s average prospect theoretic utility, average offloaded image data amount, average delay, average energy consumption, average payment and failure probability of the edge server.

In this section, we discuss the impact of the computing delay pricing $p_{t{d_i}}^{S\_ct} = \omega \cdot \varphi$, imposed by the edge server on the user’s computation offloaded strategy, where the payment factor $\omega$ is from 0.001 to 0.9. The simulation results are shown in Fig. 4. It can see that as $\omega$ increases, the average prospect theoretic utility of users gradually increases to its maximum after that slowly decreases (Fig. 4A). Specifically, when the $\omega$ is small (*i.e*., $\omega$ = 0.001), the edge server will charge a lower $p_{t{d_i}}^{S\_ct}$ and users will offload a large amount of image data (Fig. 4B), which results in lower delay (Fig. 4C) and lower payment (Fig. 4E), higher energy consumption (Fig. 4D), lower prospect theoretic utility (Fig. 4A). But the edge server will bear huge computing pressure, which causes a higher $Pr$ (Fig. 4F). When $\omega$ further increases, the edge server will charge a higher $p_{t{d_i}}^{S\_ct}$. Users are not willing to use the computing resources of the edge server, the delay and payment will gradually increase, the energy consumption and $Pr$ will reduce, a lower prospect theoretic utility again. Therefore, we need to balance the computing delay pricing to maintain the user’s high-quality experience. In addition, Figs. 4G–4I indicate the joint distribution between average utility and average delay, average energy consumption and average cost, respectively. It is clear from the regression line that the average utility shows a decreasing, increasing and decreasing trend with the increase of the three, respectively.

#### Impact of the number of users

**Figure 5:**
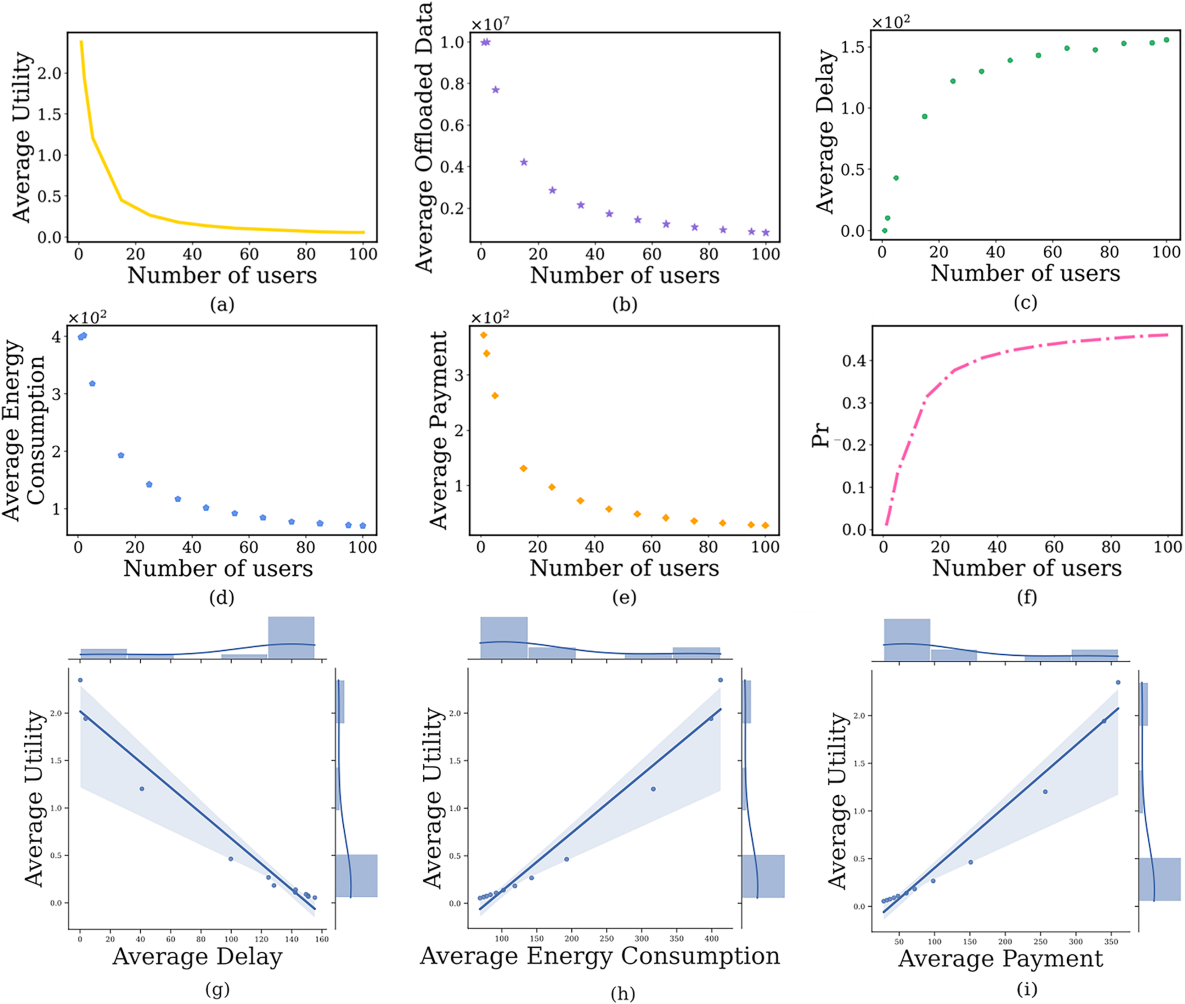
The relationship between the number of users and user’s average prospect theoretic utility, average offloaded image data amount, average delay, average energy consumption, average payment and failure probability of the edge server.

In this section, we discuss the impact of the number of users on the user’s computation offloading strategy, where the number of users is from 1 to 100. The simulation results are shown in Fig. 5. When the *N* is small (*i.e*., *N* = 1, 2, 5), the $Pr$ is very low (Fig. 5F) because the computing resources on the edge server are far greater than the needs of users. Users are inclined to offload a large amount of image data to the edge server (Fig. 5B), resulting in lower delay (Fig. 5C), higher payment (Fig. 5E), higher energy consumption (Fig. 5D), and higher prospect theoretic utility (Fig. 5A). As *N* further increases, the edge server is under more and more computing pressure. Users tend to offload a small amount of image data to the edge server, while the remaining image process on the local device, which results in higher delay, lower payment, lower energy consumption, and lower prospect theoretic utility. Similarly, Figs. 5G–5I indicate the joint distribution between average utility and average delay, average energy consumption and average cost, respectively. It is clear from the regression line that the average utility shows a decreasing, increasing and increasing trend with the increase of the three, respectively.

#### The relationship between computing delay pricing and number of users

**Figure 6:**
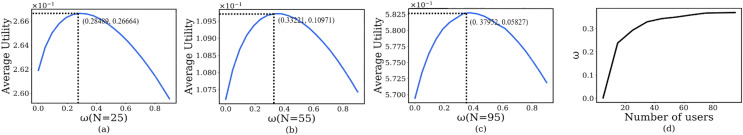
The relationship between computation delay pricing and number of users.

In this section, we discuss the relationship between computation delay pricing $p_{t{d_i}}^{S\_ct} = \omega \cdot \varphi$ and the number of users *N*. The simulation results are shown in Fig. 6.

Especially, in Figs. 6A–6C, the x-axis shows the payment factor $\; \omega$, and the y-axis shows the average PTU under *N* = 25, 55 and 95, respectively. It is noted that the average PTU of users gradually increases to its maximum with the increase of $\omega$ after that slowly decreases. Moreover, the payment factor $\omega$ is different when the maximum PTU is reached under different user’s numbers. To explore the relationship between the two, we performed the following experiments. Figure 6D shows the computation delay pricing $p_{t{d_i}}^{S\_ct} = \omega \cdot \varphi$ corresponding to the maximum utility of a different number of users. When the number of users is small (*i.e*., 1, 2), the computing pressure on the edge server is very small, so it will impose a smaller payment factor $\; \omega$, *i.e*., a lower computation delay pricing $p_{t{d_i}}^{S\_ct}$. As the number of users further increases, the edge server is under more and more computing pressure. To reduce the possibility of failure, the edge server will control the number of users by increasing $p_{t{d_i}}^{S\_ct}$. Since that *N* = 85, there is no change in both convergence speed and convergence result.

#### Impact of the multi-objective weight coefficients

**Figure 7:**
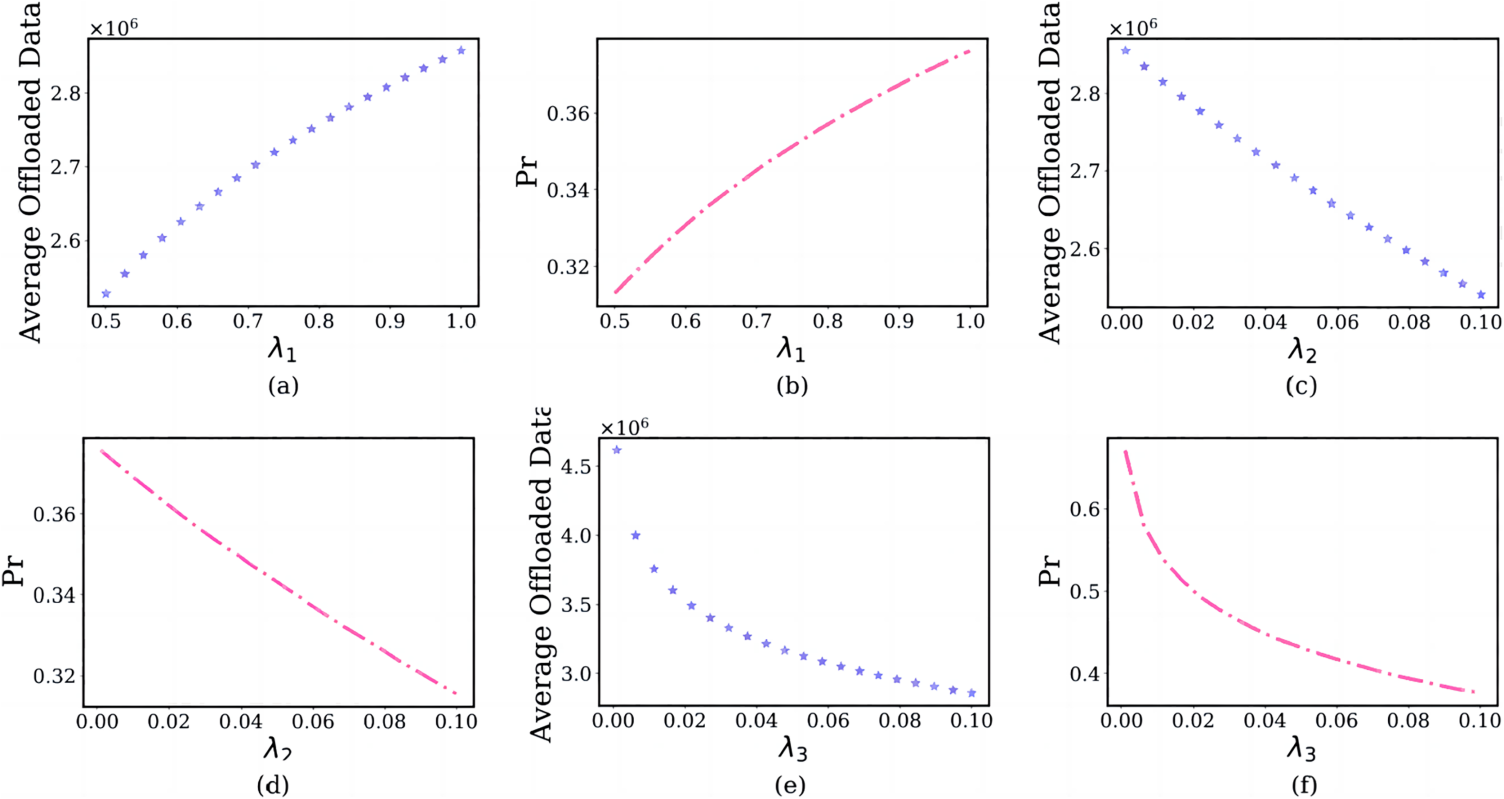
The relationship between multi-objective weights and user’s average offloaded image data amount and failure probability of the edge server.

In this section, we discuss the impact of the multi-objective weight coefficients on the user’s computation offloaded strategy, where multi-objective weight coefficients ${\lambda _1} > {\lambda _2} > {\lambda _3}$. The simulation results are shown in Fig. 7.

In the subfigure of Figs. 7A and 7B, the x-axis shows the delay weight ${\lambda _1}$, and the y-axis shows the user’s average offloaded image data amount and failure probability of the edge server in each ${\lambda _1}$, where ${\lambda _2} = 0.1$, ${\lambda _3} = 0.01$. As the ${\lambda _1}$ increases from 0.5 to 1, users are inclined to offload an enormous amount of image data (Fig. 7A). The reason is that the scenario set in this article is a delay-sensitive task, and users choose a larger ${\lambda _1}$ rather than smaller ${\lambda _1}$. This will bring tremendous pressure to the edge server and increase the failure probability (Fig. 7B).

In the subfigure of Figs. 7C and 7D, the x-axis shows the energy consumption weight ${\lambda _2}$, and the y-axis shows the user’s average offloaded image data amount and failure probability of the edge server in different ${\lambda _2}$, where ${\lambda _1} = 1$, ${\lambda _3} = 0.01$. The reason is that users are insensitive to energy consumption for delay-sensitive tasks. As the ${\lambda _2}$ increases from 0.001 to 0.1, a fewer images will be offloaded (Fig. 7C), which reduces the failure probability (Fig. 7D).

In the subfigure of Figs. 7E and 7F, the x-axis shows the payment weight ${\lambda _3}$, and the y-axis shows the user’s average offloaded image data amount and failure probability of the edge server in different ${\lambda _3}$, where ${\lambda _1} = 1$, ${\lambda _2} = 0.1$. For the same reason, users are also relatively insensitive to payments. As the ${\lambda _3}$ increases from 0.001 to 0.1, a little image will be offloaded (Fig. 7E) and the failure probability of the edge server also will reduce (Fig. 7F).

#### Impact of the prospect theoretic parameters

**Figure 8:**
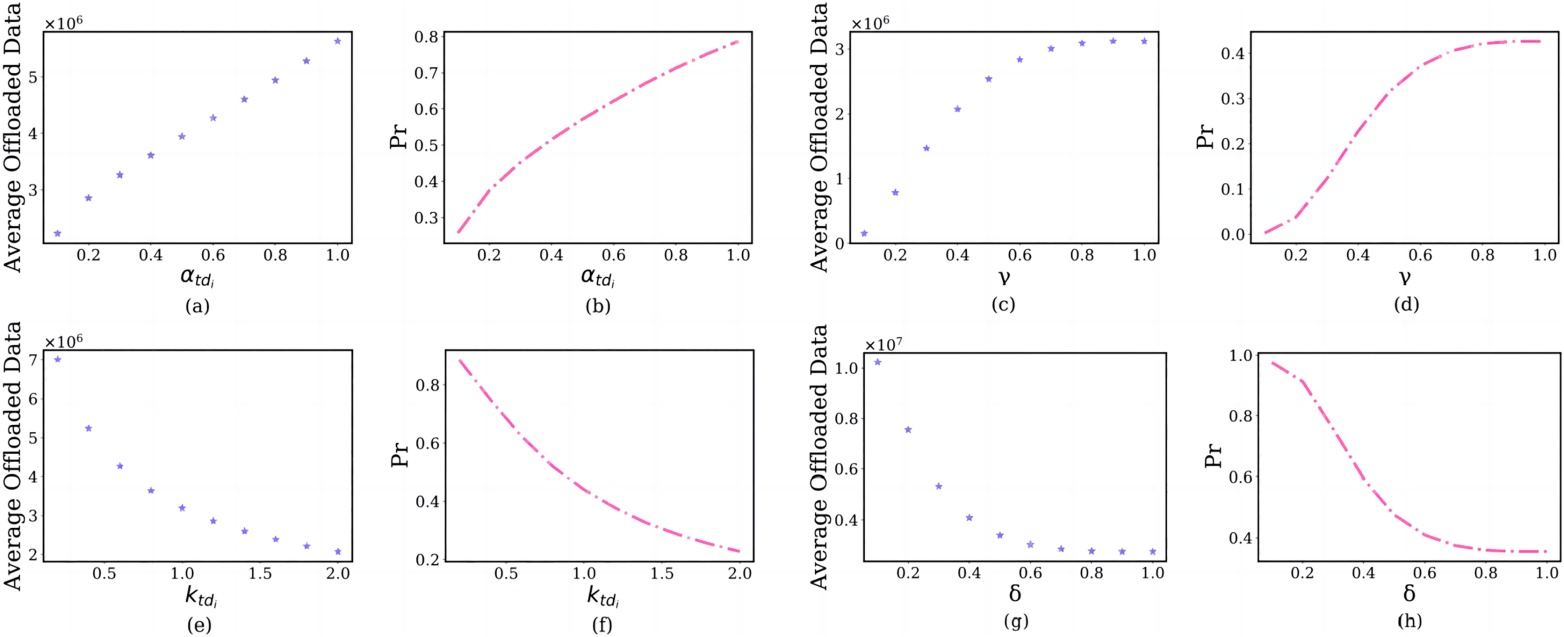
The relationship between prospect theoretic parameters and user’s average offloaded image data amount and failure probability of the edge server.

In this section, we discuss the impact of the prospect theoretic parameters, including risk attitude ${\alpha _{t{d_i}}}$, gain attitude $\gamma$, loss aversion ${k_{t{d_i}}}$ and loss attitude $\delta$ on the user’s offloading strategy. The simulation results are shown in Fig. 8.

In the subfigure of Figs. 8A and 8B, the x-axis shows the ${\alpha _{t{d_i}}}$, and the y-axis shows the user’s average offloaded image data amount and failure probability of the edge server in each ${\alpha _{t{d_i}}}$. As the risk attitude ${\alpha _{t{d_i}}}$ increases from 0 to1, users are inclined to offload a larger amount of image data (Fig. 8A). The reason is that they will choose larger gains, not smaller gains. As the ${\alpha _{t{d_i}}}$ increases, the failure probability of the edge server will also increase (Fig. 8B). For the same reason, As the gain attitude $\gamma$ increases from 0.1 to 1, users will have a larger average offloaded image data amount (Fig. 8C) and larger failure probability of the edge server (Fig. 8D).

In the subfigure of Figs. 8E and 8F, the x-axis shows the ${k_{t{d_i}}}$, and the y-axis shows the user’s average offloaded image data amount and failure probability of the edge server in each ${k_{t{d_i}}}$. As the loss aversion ${k_{t{d_i}}}$ increases from 0.1 to 2, they offload fewer images to the edge server (Fig. 8E). The reason is that the ${k_{t{d_i}}}$ and user’s loss aversion are positively correlated. This will lead to the failure probability of the edge server decreasing (Fig. 8F). For the same reason, As the loss attitude $\delta$ increases from 0 to 1, the user will have a lower average offloaded image data amount (Fig. 8G) and larger failure probability of the edge server (Fig. 8H).

### Convergence analysis

**Figure 9:**
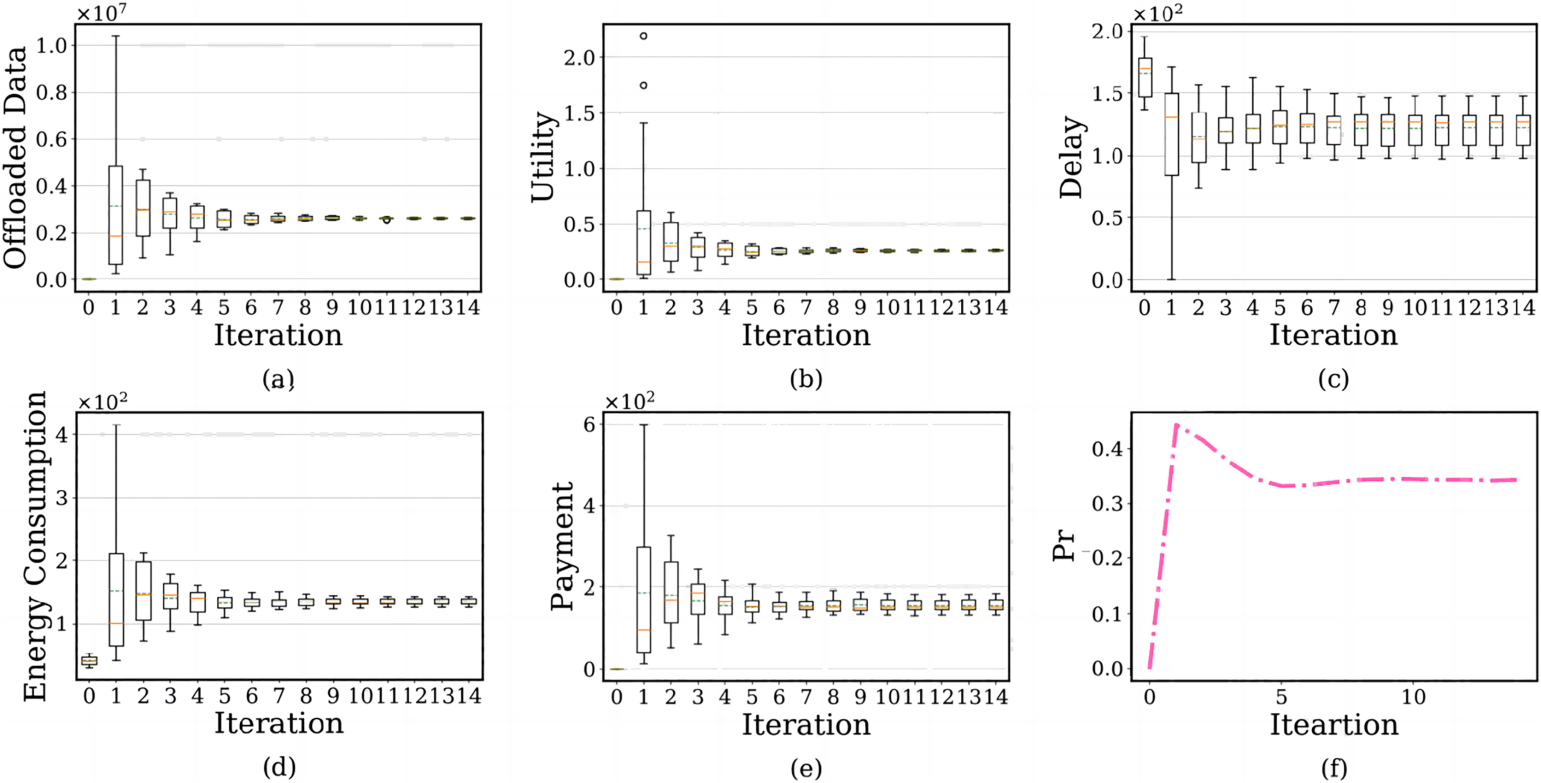
The relationship between the number of iterations and each user’s offloaded image data amount, prospect theoretic utility, delay, energy consumption, payment and failure probability of the edge serve.

We evaluate the convergence of the BRD-CO algorithm. The simulation results are shown in Fig. 9. In the subfigure, the x-axis shows the number of iterations, and the y-axis shows each user’s offloaded image data amount, PTU, delay, energy consumption, payment and failure probability of the edge server.

There are two lines on the above and below of the boxes, which represent the maximum and minimum offloaded amounts. The red solid line is the median of the amount of offloaded image data, representing the average level, and the green dotted line represents the average level of offloaded image data after using the BRD-CO algorithm. Figures 9A–9E shows the convergence of each user’s offloaded image data amount, prospect theoretic utility, delay, energy consumption and payment after the image is offloaded to the edge server. The results show that the BRD-CO algorithm converged faster with fewer iterations.

**Figure 10:**
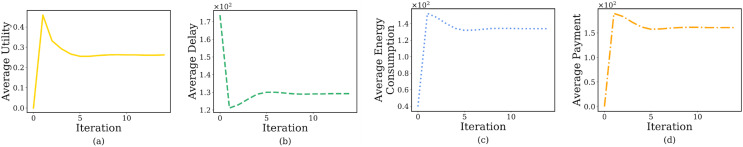
The relationship between iteration number and user’s average prospect theoretic utility, average delay, average energy consumption and average payment.

Figure 10 shows the user’s average prospect theoretic utility, average delay, average energy consumption and average payment with the increase of iteration number. Specifically, each user will offload a large image on the edge server instead of the terminal device when *ite* = 1 (Figs. 9A and 10A). This behavior is highly likely to trigger the overuse of computing resources, which makes the $Pr$ increase dramatically (Fig. 9F). Therefore, the edge servers will curb this occurrence by charging users for higher energy consumption (Fig. 10C) and higher payment (Fig. 10D). Surprisingly, the algorithm will reach the lowest delay (Fig. 10B) because of the high computing power of the edge server and performing massive diagnosis tasks.

Considering the failure probability of the edge server, as *ite* increases from 2 to 5, the convergence speed of the BRD-CO algorithm is increase. It will lead to the rapid decrease of image data offloaded amount (Fig. 9A), prospect theoretic utility value (Fig. 10A), energy consumption (Fig. 10C) and payment (Fig. 10D) and the rapid increase of delay (Fig. 10B). As it further increases from 5 to 14, there is no change in both convergence speed and convergence result.

### Method comparison

In this session, to evaluate the proposed BRD-CO method, we provide a comparative study between the proposed method with the following four benchmarks and four heuristic methods.

(1) Local computing (denoted by L. Comp.): all tasks are executed on the user terminal without offloading.

(2) Full offloading (denoted by F. Offl.): all tasks are executed on the edge server.

(3) Random offloading (denoted by R. Offl.): each task is randomly offloaded to the user terminal or edge server.

(4) Greedy offloading (denoted by G. Offl.): find the best offloading location for each task by selecting the current optimal solution each time.

(5) Particle swarm optimization-based offloading (denoted by PSO. Offl.) (Yuan et al., 2022): PSO simulates the foraging behavior of a flock of birds, using collaboration and information sharing among individuals in the flock to find the best decision to determine the offloading position of each task.

(6) Differential evolution-based offloading (denoted by DE. Offl.) (Hussain & Beg, 2021): DE simulates biological evolution by iterating repeatedly so that those individuals that are adapted to the environment are retained and the offloading position of each task is determined.

(7) Simulate anneal-based offloading (denoted by SA. Offl.) (Li, 2021): SA algorithm draws on the similarities, which exist between the annealing process of solids in statistical physics and general combinatorial optimization problems, to find the execution position of each task.

(8) Ant colony optimization-based offloading (denoted by ACO. Offl.) (Lin, Pankaj & Wang, 2018): each ant in the ACO algorithm uses pheromones to search simultaneously and independently at multiple points in the problem space, eventually finding the offloading position for each task.

Since the optimization goal of this article is to maximize the user’s prospect theoretic utility, we choose average utility as the performance metric. To eliminate the stochastic introduced by the heuristic algorithms, we conduct 50 runs and used the mean and standard deviation of PTU value as the final result. Table 5 and Table 6 in the Supplemental Information show the results of various heuristic algorithms for different payment factor $\omega$ and different numbers of users, respectively.

**Figure 11:**
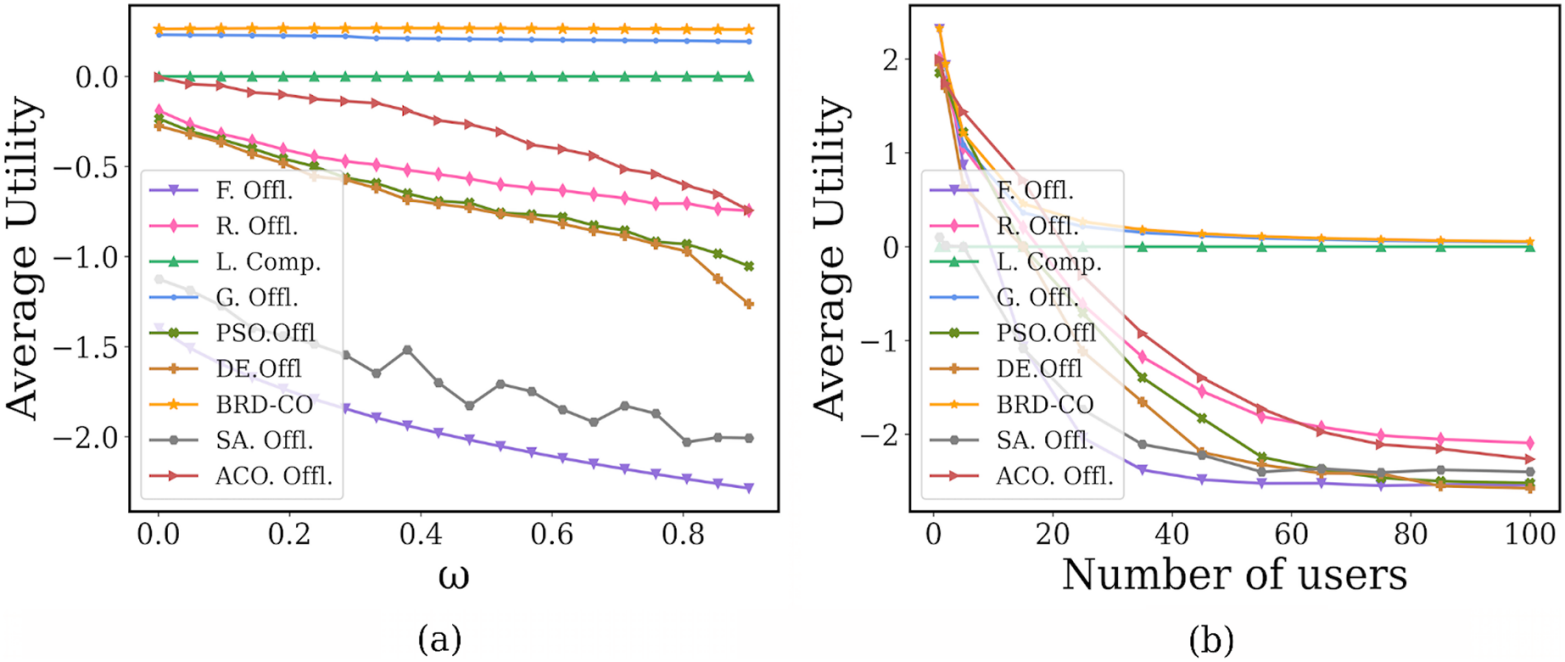
Performance under different payment factors.

In Fig. 11A, as the payment factor $\omega$ increases, the proposed BRD-CO algorithm can always maintain a higher average utility when compared with the benchmark methods. When the $\omega$ is small (*i.e*., $\omega$ = 0.001), the maximum average utility can be achieved by most methods, but it is maximum in our method. As its future increases from 0.001 to 0.9, the average utility of our proposed BRD-CO algorithm decreases by 1.14%, while those of the benchmark methods (expect L. Offl.) decrease by at least 16.4%. This implies that as the computing delay pricing $p_{t{d_i}}^{S\_ct} = \omega \cdot \varphi$ increases, the average utility of the proposed algorithm decreases less dramatically than other methods. The reason is that when the payment factor $\omega$ is larger, the users are inclined to offload fewer image, resulting in the average utility decreasing slowly.

In Fig. 11B, the proposed BRD-CO algorithm always achieves a higher average utility than the benchmark methods, especially when the number of users is small. As the number of users increases, the average utility of each algorithm (expect L. Offl.) decreases and gradually converges. The reason is that when the number of users is larger, the potential pressure at the edge servers will be increased. When the number of users is large enough, each user has very little offload. In addition, in this article, a marginal decrease of less than 0.005 in average utility is called convergence. From Fig. 11B, we can find that only the BRD-CO algorithm can converge when the number of users is 45. For G. Offl., R. Offl., F. Offl., PSO. Offl., DE. Offl., SA. Offl., and ACO. Offl., convergence is achieved at 50, 85, 55, 85, 85, 75, and 100, respectively (expect L. Offl.). When the BRD-CO reaches convergence, the average utility is 0.138, which is greater than G. Offl. (0.115), L. Offl. (0), R. Offl. (−2.052), F. Offl. (−2.523), PSO. Offl. (−2.500), DE. Offl. (−2.551), SA. Offl (−2.39) and ACO (−2.265). Therefore, compared with other methods, our proposed BRD-CO algorithm has a faster convergence speed and higher average utility.

Statistical test is an effective way to evaluate the performance of algorithms. In this article, the Wilcoxon rank sum test (Derrac et al., 2011) is adopted as a non-parametric statistical test that returns a *P*-value that verifies the significant level difference between the two algorithms. It is worth noting that an algorithm is statistically different when the *P*-value is less than 0.05. The *P* values obtained from formula (21) under different payment factor and different number of users are shown in Table 7 of the Supplemental Information. By evaluating the comparison between BRD-CO and the other eight algorithms, it is clearly understood that only one of the 16 *P*-values exceeds 0.05, which reflects the statistical superiority of BRD-CO.

## Conclusions

In this article, we propose a multi-user multi-objective computation offloading for medical image diagnosis, which can play a significant role in the medical image cloud. Prior computation offloading strategies ignored payment required to perform computation tasks and a user’s risk awareness. To reflect the real communication and computing environment, we consider a more realistic optimization of multi-objective. Specifically, to maximize the prospect theoretic utility of each user by considering delay, energy consumption, payment and user’s risk awareness, we design a low-complexity BRD-CO algorithm. The algorithm can quickly converge to NE point and obtain an optimal computation offloading strategy for each user in a distributed manner. The parameter influence analysis of the BRD-CO algorithm is verified by five aspects. The simulation results show that when compared with four benchmarks and four heuristic algorithms, our proposed BRD-CO algorithm can guarantee a higher user’s prospect theoretic utility and a faster convergence speed. The benefit is especially significant when the diagnosis tasks are delay-sensitive and the resources of terminal devices are limited.

It is worth noting that the medical tasks studied in this article are coarse-grained, but some medical tasks can be more fine-grained. Therefore, our future research work focuses on task dependencies. For diagnostic tasks based on radiomics model, the association relationships between sub-modules (*i.e*., subtasks) within the model have a large impact on the task offloading problem. Therefore, we intend to use the recurrent neural network to model the dependencies between subtasks. Meanwhile, the high-quality task offloading strategy is learned by continuous ‘trial and error’ through deep reinforcement learning.

## Supplemental Information

10.7717/peerj-cs.1239/supp-1 Supplemental Information 1 Simulation code for computation offloading algorithm based on the Best Response Dynamics.

10.7717/peerj-cs.1239/supp-2 Supplemental Information 2 Theorem proof.

## Additional Information and Declarations

### Data Availability

The following information was supplied regarding data availability: The data is available at Zenodo: lq-github. (2023). lq-github/BRD-CO: First release of BRD-CO (v1.0.0). Zenodo. https://doi.org/10.5281/zenodo.7505089.
