# Supplementary figures and images for: Multi-user multi-objective computation offloading for medical image diagnosis
Source: PeerJ Comput Sci. 2023 Mar 8;9:e1239. doi: 10.7717/peerj-cs.1239 (PMC10280585; doi:10.7717/peerj-cs.1239)

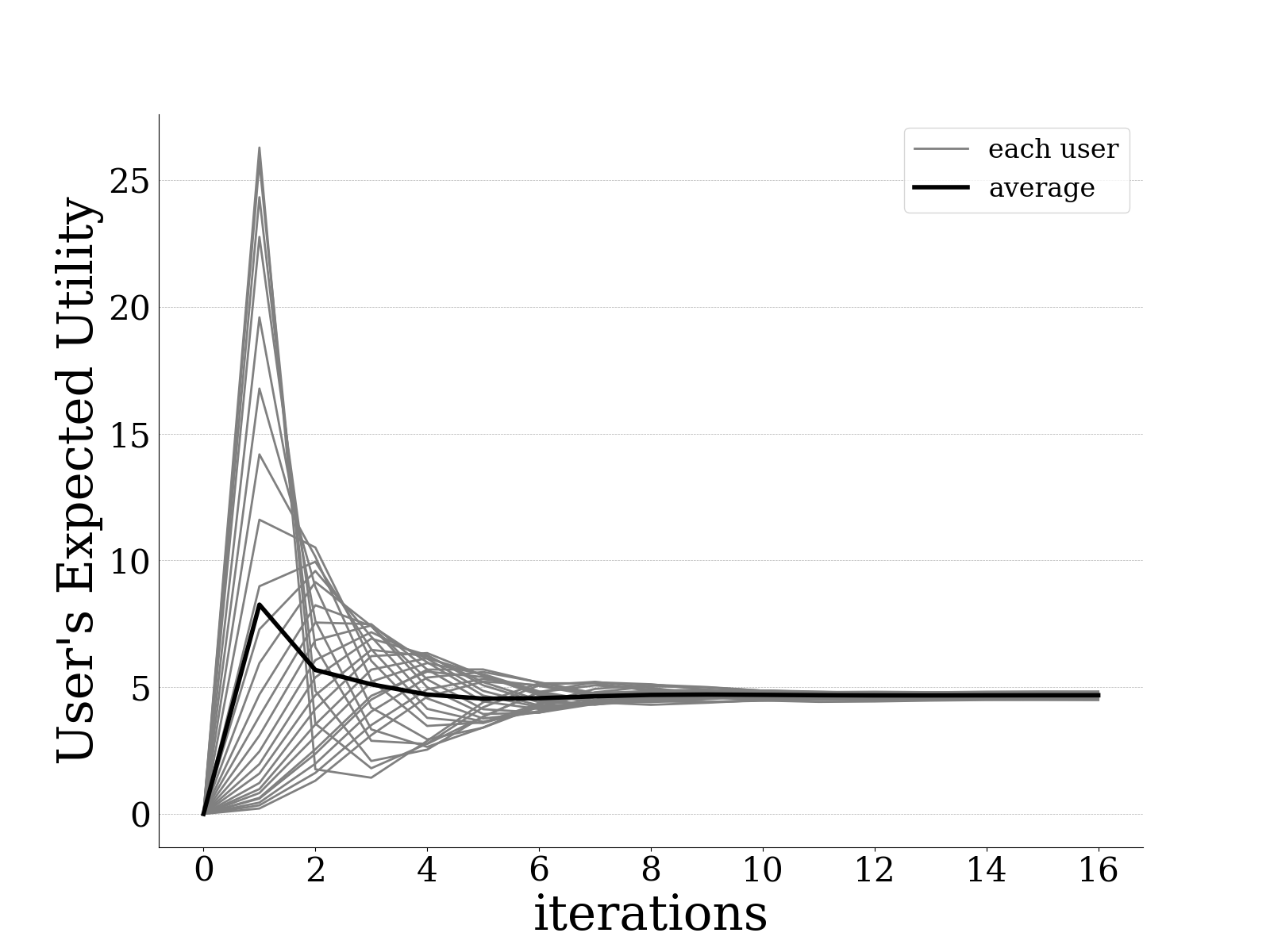

Supplement: Supplemental Information 1 [file peerj-cs-09-1239-s001.zip › Computation offloading algorithm based on the Best Response Dynamics/unused/expected_utility.png]

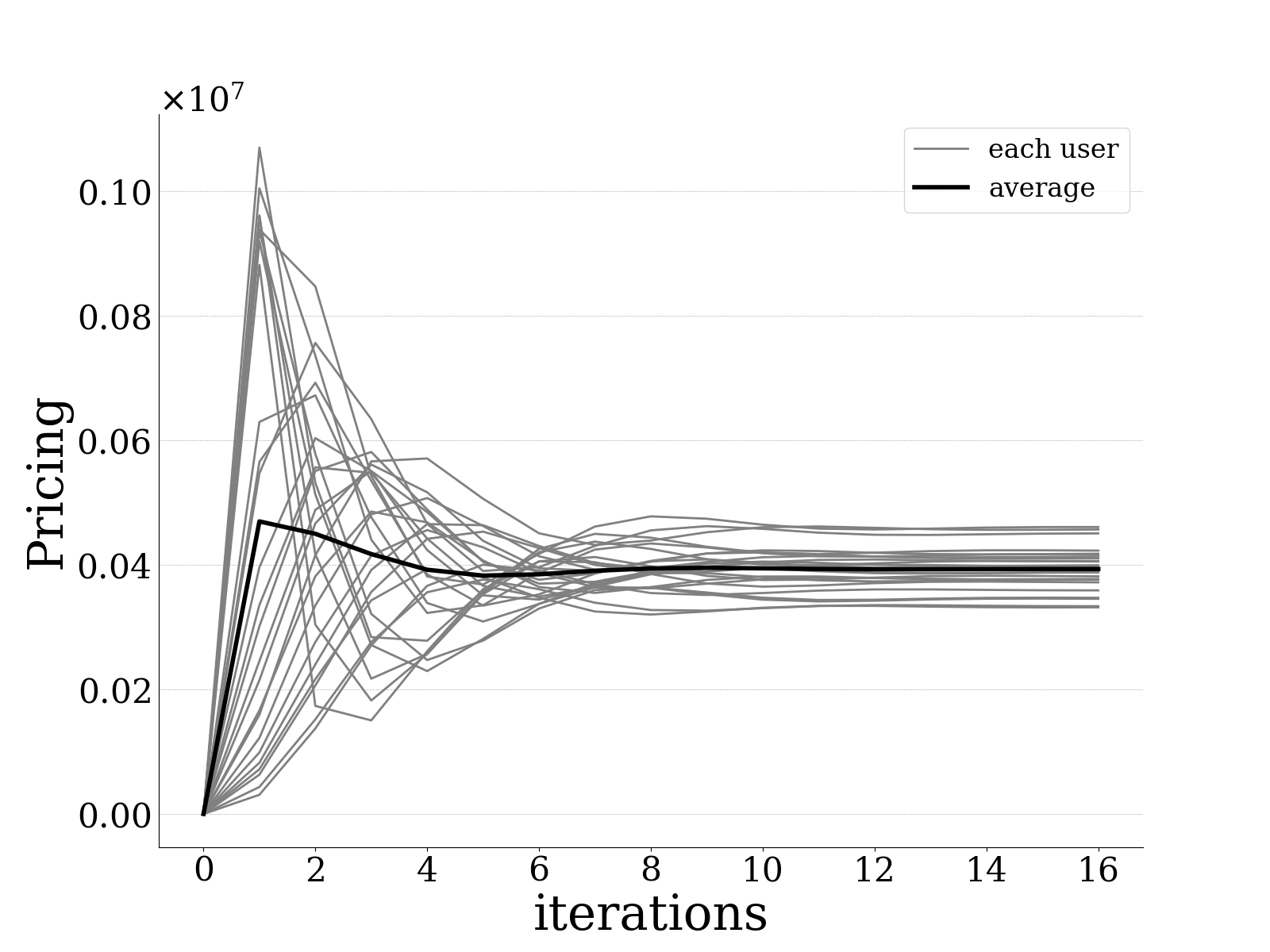

Supplement: Supplemental Information 1 [file peerj-cs-09-1239-s001.zip › Computation offloading algorithm based on the Best Response Dynamics/unused/pricing.png]

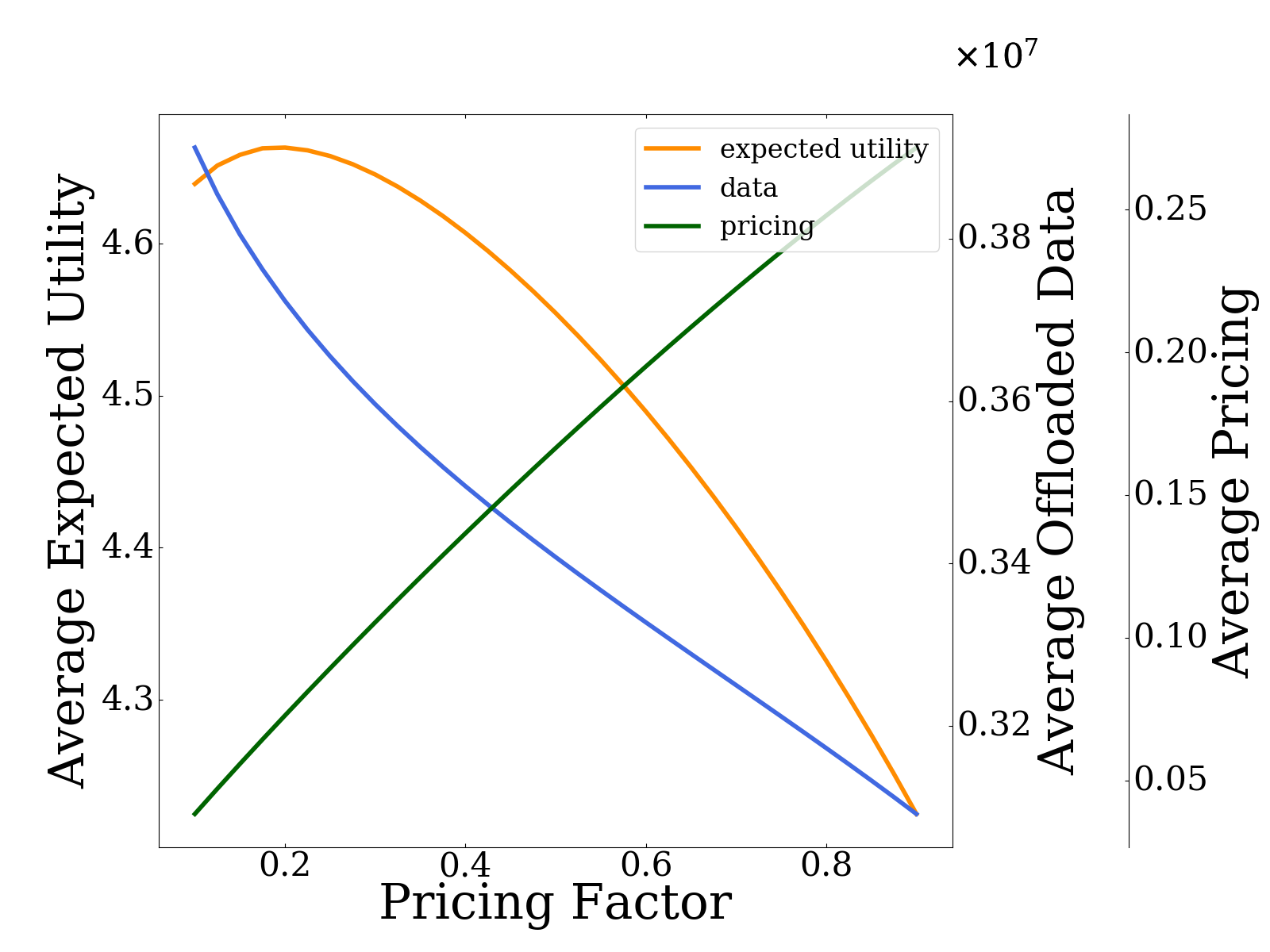

Supplement: Supplemental Information 1 [file peerj-cs-09-1239-s001.zip › Computation offloading algorithm based on the Best Response Dynamics/unused/different_c.png]

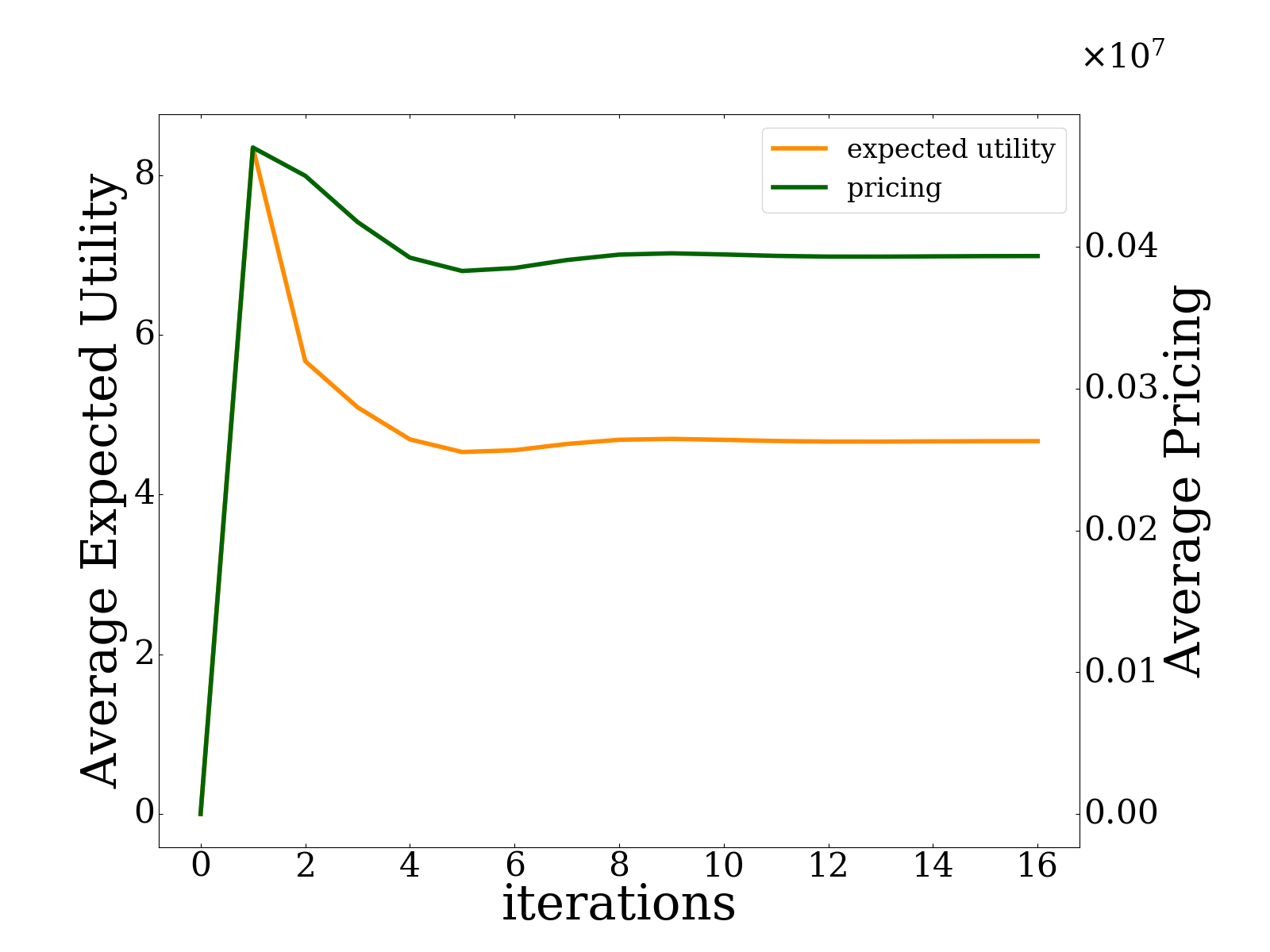

Supplement: Supplemental Information 1 [file peerj-cs-09-1239-s001.zip › Computation offloading algorithm based on the Best Response Dynamics/unused/expected_utility_and_pricing.png]

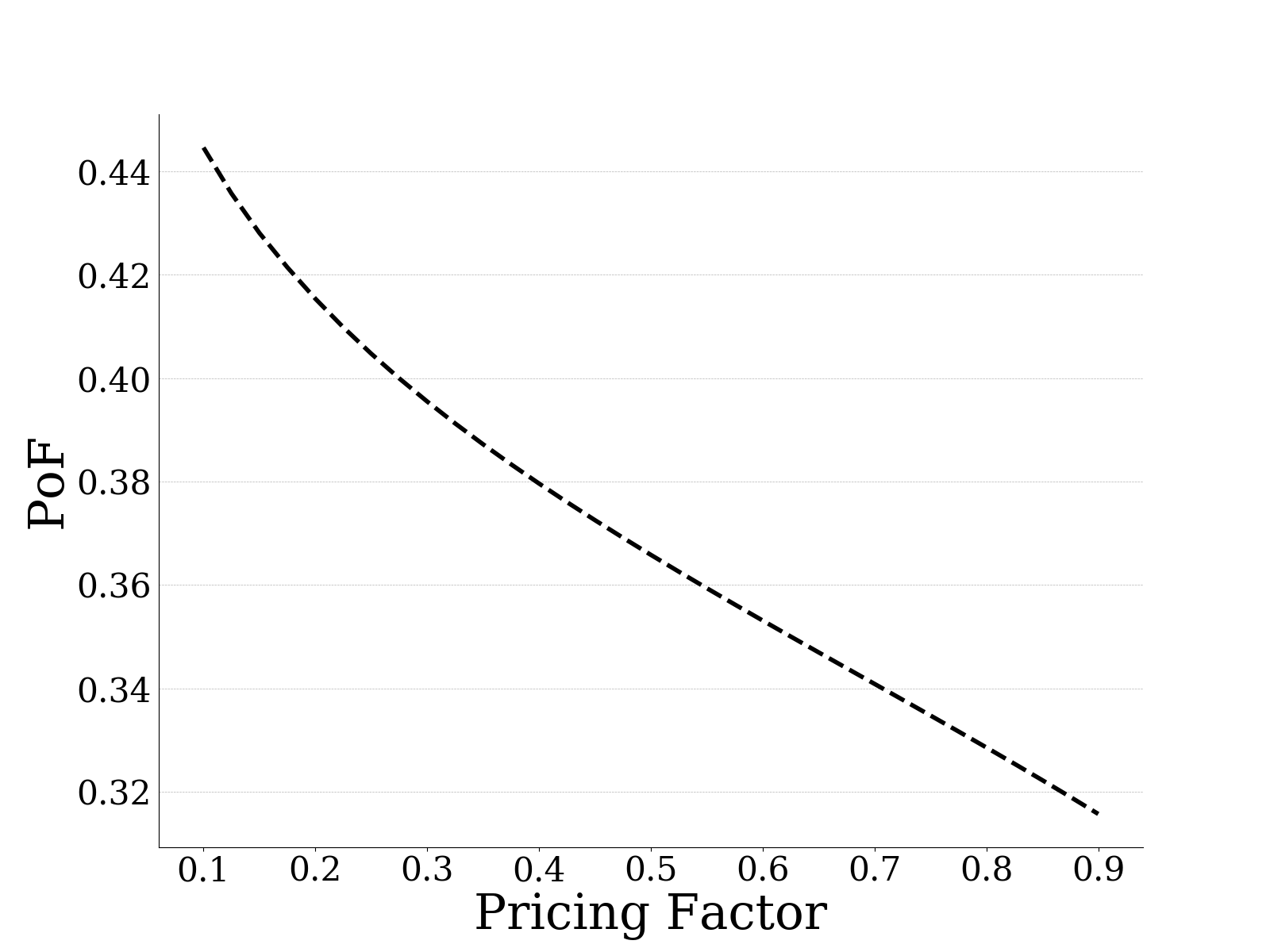

Supplement: Supplemental Information 1 [file peerj-cs-09-1239-s001.zip › Computation offloading algorithm based on the Best Response Dynamics/unused/PoF_vs_cost.png]

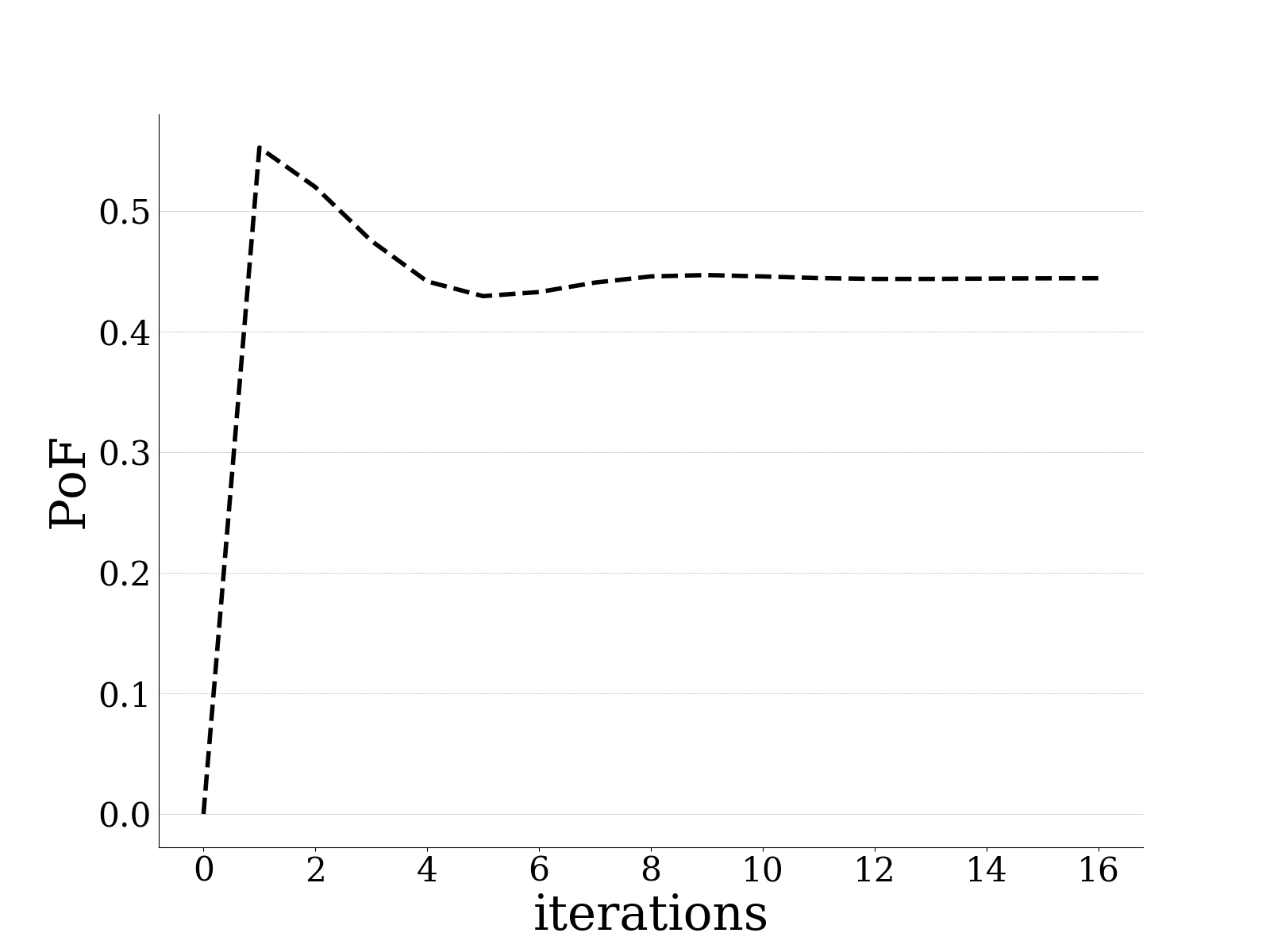

Supplement: Supplemental Information 1 [file peerj-cs-09-1239-s001.zip › Computation offloading algorithm based on the Best Response Dynamics/unused/PoF.png]

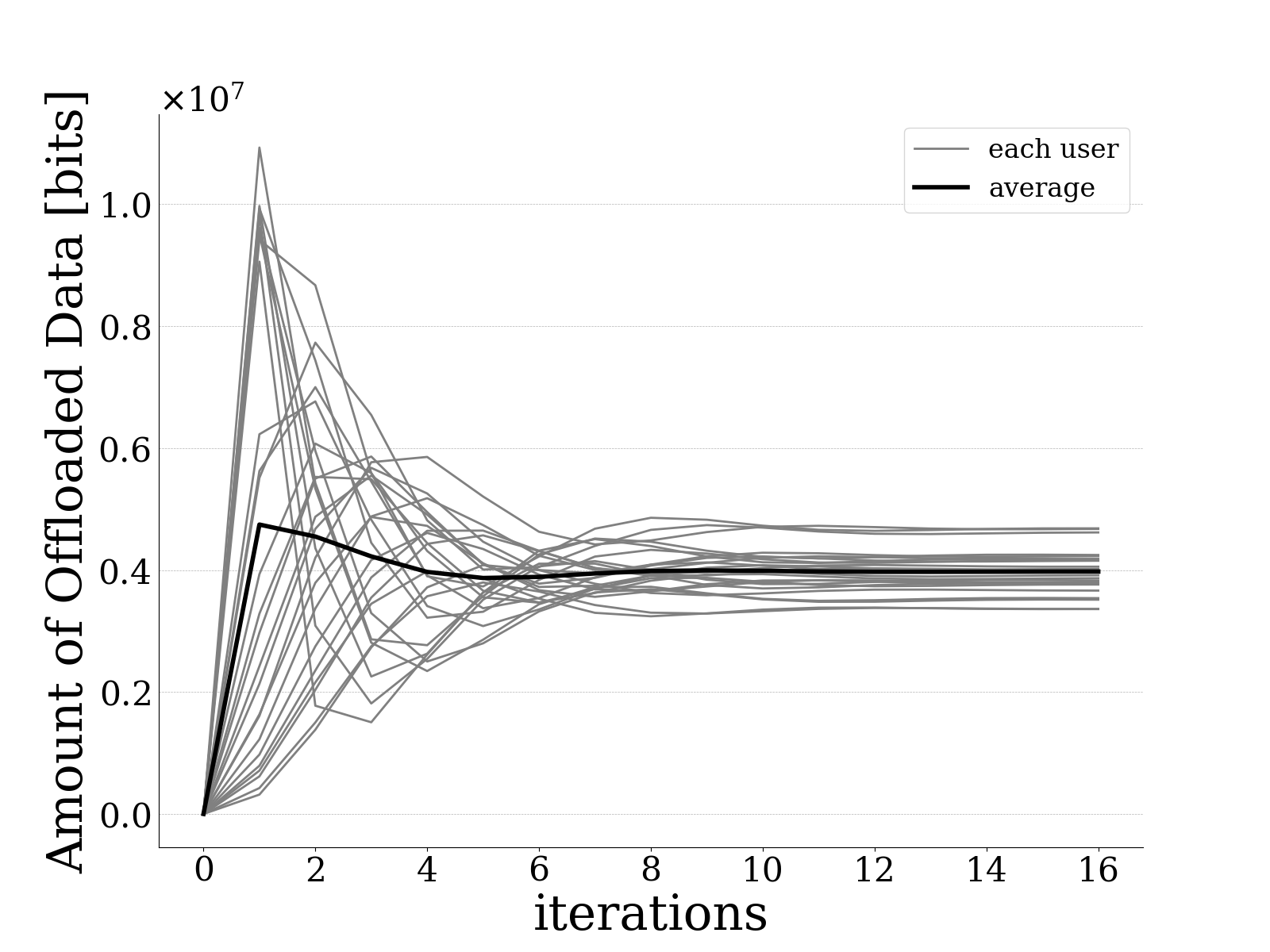

Supplement: Supplemental Information 1 [file peerj-cs-09-1239-s001.zip › Computation offloading algorithm based on the Best Response Dynamics/unused/b_converging.png]

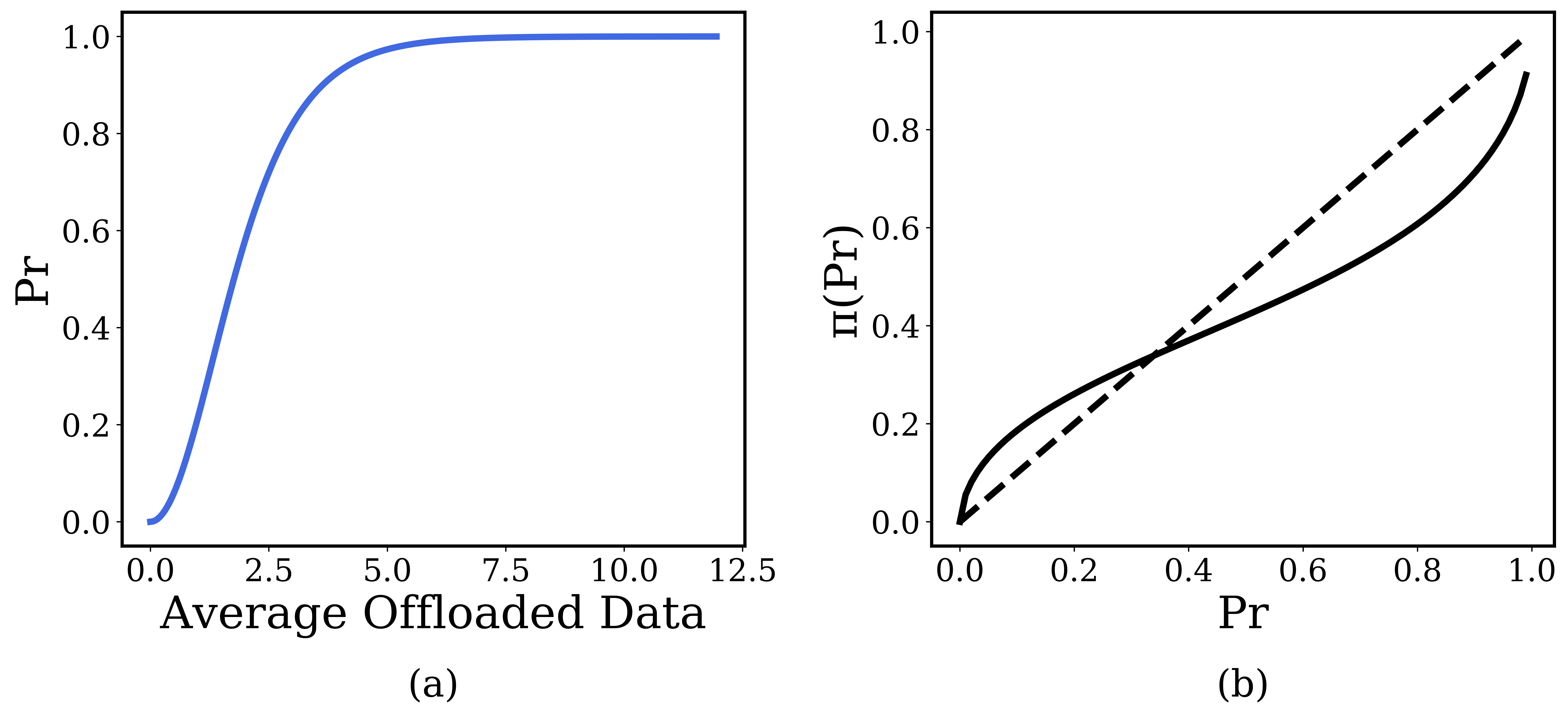

Supplement: Supplemental Information 1 [file peerj-cs-09-1239-s001.zip › Computation offloading algorithm based on the Best Response Dynamics/plots/PoF_div_Pof_weight.png]

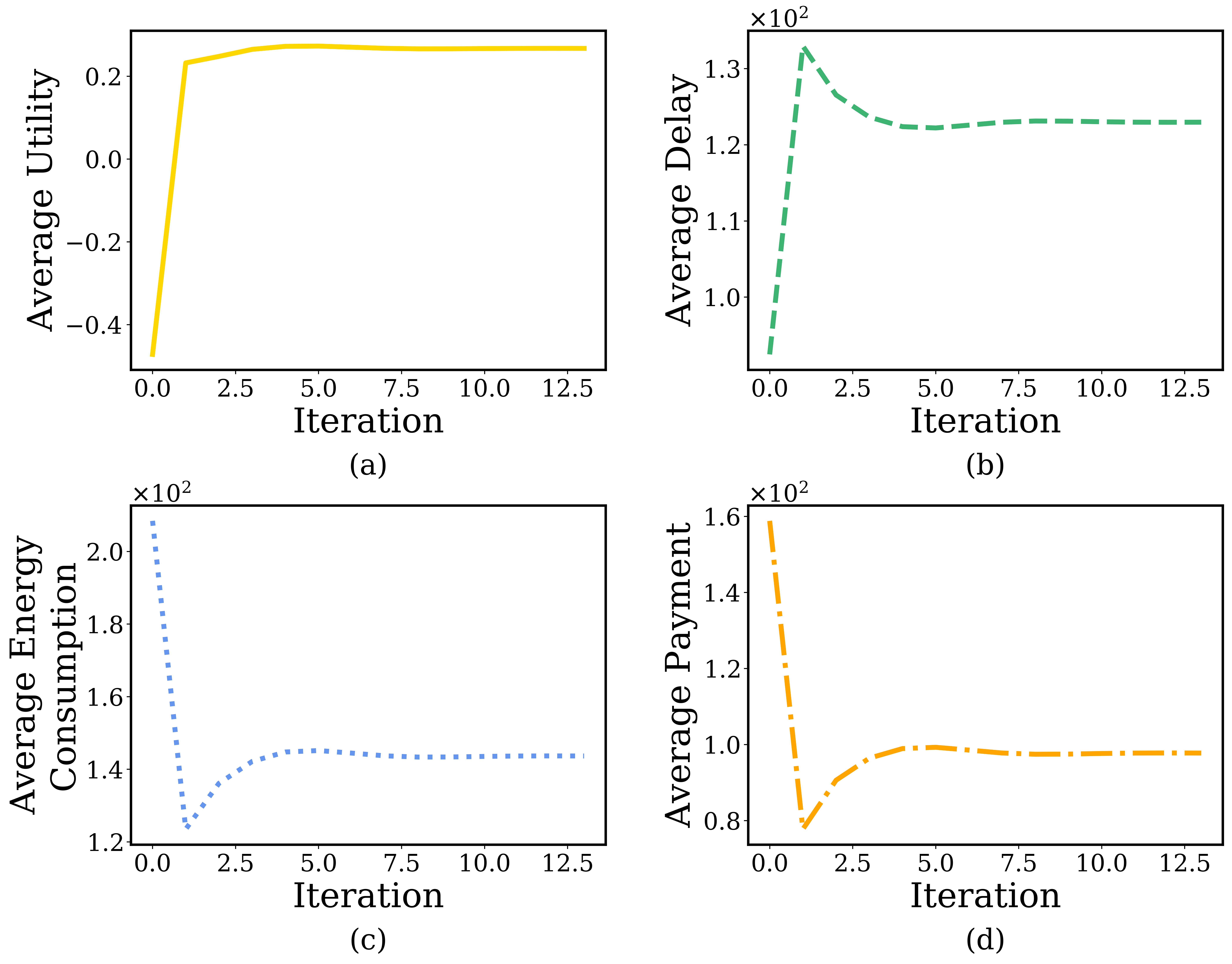

Supplement: Supplemental Information 1 [file peerj-cs-09-1239-s001.zip › Computation offloading algorithm based on the Best Response Dynamics/plots/iterations_E_T_E_P.png]

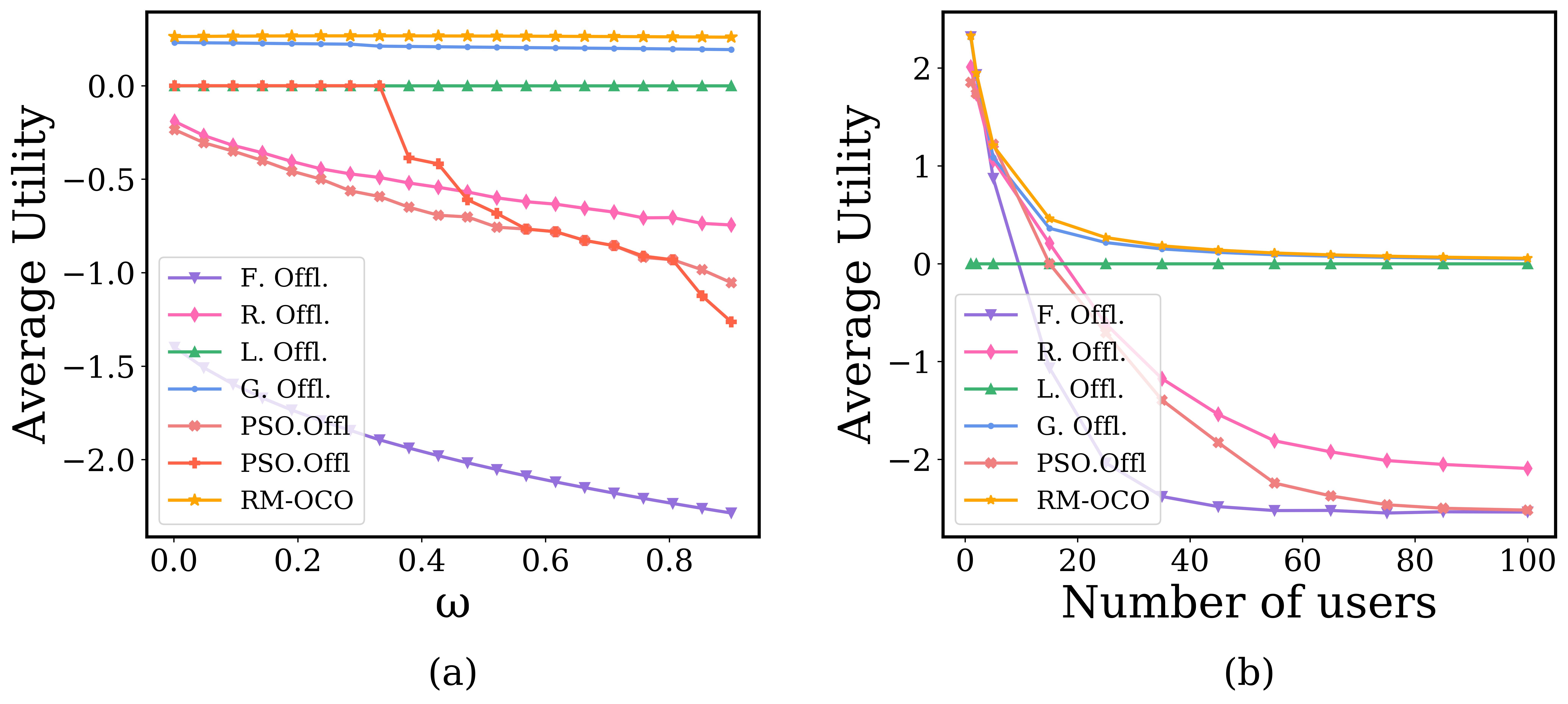

Supplement: Supplemental Information 1 [file peerj-cs-09-1239-s001.zip › Computation offloading algorithm based on the Best Response Dynamics/plots/comparion_method1.png]
