# Supplementary material for: Multi-user multi-objective computation offloading for medical image diagnosis
Source: PeerJ Comput Sci. 2023 Mar 8;9:e1239. doi: 10.7717/peerj-cs.1239 (PMC10280585; doi:10.7717/peerj-cs.1239)
Supplement: Supplemental Information 2 [file peerj-cs-09-1239-s002.docx]

**Appendix**

***Definition* 1**. Computation offloading () model is 8-tuple:

, where:

1. is the finite set of user terminal devices. denotes the device used by the i-th user. refers to the total number of users, .
2. is the finite set of diagnosis tasks. denotes the computation task of .
3. is the finite set of offloading proportions. denotes the offloading proportion of .
4. is task computational complexity, i.e., Required CPU cycles for computing per bit of diagnosis task . The computational complexity of each task is the same.
5. is the expected profits for processing per bit of the diagnosis task.
6. is the finite set of local computing parameters, where:
7. is the finite set of the computational capability. denotes the computational capability of ,
8. is the finite set of the energy coefficient. denotes the consumed energy per CPU cycle of ,
9. is the finite set of the computation delay locally. denotes the computation delay required by to process locally,
10. is the finite set of the computation energy consumption locally. denotes the computation energy consumption required by to process locally.
11. is the finite set of full offloading parameters, where
12. is the computational capability of the edge server,
13. is the energy coefficient denoting the consumed energy per CPU cycle of the edge server,
14. is the finite set of computation delay pricing. denotes the computation delay pricing per second by the edge server imposed on ,
15. is the finite set of transmission power. denotes the transmission power between and the edge server,
16. is the finite set of the image transmission rate. denotes the transmission rate between and the edge server,
17. is the finite set of the total delay on the edge server. denotes the total delay required by to process on the edge server. , where is the finite set of the computation delay on the edge server. denotes the computation delay required by to process on the edge server. is the finite set of the transmission delay on the edge server. denotes the transmission delay required by to process on the edge server,
18. is the finite set of the total energy consumption on the edge server. denotes the total energy consumption required by to process on the edge server. , where is the finite set of the computation energy consumption on the edge server. denotes the computation energy consumption required by to process on the edge server. is the finite set of the transmission energy consumption on the edge server. denotes the transmission energy consumption needed by to process on the edge server,
19. is the finite set of the payment. denotes the computation delay cost required by to process on the edge server.
20. is the finite set of partial offloading parameters, where
21. is the finite set of the total delay in partial offloading. denotes the delay required by to process in partial offloading,
22. is the finite set of the total energy consumption in partial offloading. denotes the energy consumption required by to process in partial offloading,
23. is the finite set of the payment in partial offloading. denotes the computation delay cost required by to process in partial offloading.

**Theorem 1**. The is an Exact Potential Game and has at least one Nash Equilibrium point .

***Proof***. we define the exact potential function as follows

(1)

Given two different sets of strategies and , only if the offloading strategy of the terminal device is changed unilaterally from to , then the variation of user’s prospect theoretic utility is as follows

(2)

The variation of the exact potential function is as follows

(3)

Since the above formula satisfies the condition of the EPG, the NCG is an Exact Potential Game, and has at least one Nash Equilibrium point .

Table 5 The results of various heuristic algorithms for different number of users

| **number**  **of user** |  | **PSO. Offl.** | **DE. Offl.** | **SA. Offl.** | **ACO. Offl.** | **number**  **of user** |  | **PSO. Offl.** | **DE. Offl.** | **SA. Offl.** | **ACO. Offl.** |
| --- | --- | --- | --- | --- | --- | --- | --- | --- | --- | --- | --- |
| **N=1** | **ave**  **std** | 1.8534  2.87e-1 | 1.9462  2.45e-1 | 0.101  0.0001 | 1.9967  0.0001 | **N=45** | **ave**  **std** | -1.8286  3.54e-2 | -2.1924  4.86e-1 | -2.2216  0.0728 | -1.3969  0.1002 |
| **N=2** | **ave**  **std** | 1.7386  4.68e-2 | 1.6941  3.70e-2 | 0.0113  0.0002 | 1.7386  0.0002 | **N=55** | **ave**  **std** | -2.2427  4.73e-2 | -2.3243  1.81e-1 | -2.3651  0.0585 | -1.7278  0.0468 |
| **N=5** | **ave**  **std** | 1.2211  1.14e-1 | 0.6397  5.41e-2 | 0.001  0.0061 | 1.4359  0.0064 | **N=65** | **ave**  **std** | -2.3739  2.12e-2 | -2.4150  5.14e-2 | -2.3794  0.0529 | -1.9737  0.0447 |
| **N=15** | **ave**  **std** | -3.29e-6  1.47e-6 | 6.05e-6  7.51e-6 | -1.084  0.0631 | 0.7094  0.0249 | **N=75** | **ave**  **std** | -2.4644  1.35e-2 | -2.4210  5.31e-2 | -2.3998  0.0441 | -2.1093  0.0378 |
| **N=25** | **ave**  **std** | -0.7058  3.03e-1 | -1.1153  2.03e-4 | -1.735  0.0978 | -0.3070  0.0934 | **N=85** | **ave**  **std** | -2.5002  8.23e-3 | -2.5513  3.79e-2 | -2.4006  0.0450 | -2.1547  0.0409 |
| **N=35** | **ave**  **std** | -1.3942  7.34e-2 | -1.6552  9.71e-2 | -2.1079  0.0433 | -0.9247  0.0513 | **N=100** | **ave**  **std** | -2.5191  6.15e-3 | -2.5753  2.94e-2 | -2.4063  0.0397 | -2.2656  0.0252 |

| **payment factor** |  | **PSO. Offl.** | **DE. Offl.** | **SA. Offl.** | **ACO. Offl.** | **payment factor** |  | **PSO. Offl.** | **DE. Offl.** | **SA. Offl.** | **ACO. Offl.** |
| --- | --- | --- | --- | --- | --- | --- | --- | --- | --- | --- | --- |
| **=0.001** | **ave**  **std** | -0.2352  7.16e-6 | -0.2755  2.14e-5 | -1.1258  0.0629 | -0.0054  0.0198 | **=0.5214** | **ave**  **std** | -0.7018  3.33e-1 | -0.7300  1.77e-4 | -1.8273  0.0188 | -0.2667  0.1112 |
| **=0.0483** | **ave**  **std** | -0.3054  4.21e-6 | -0.3222  2.17e-5 | -1.1880  0.1327 | -0.0431  0.0196 | **=0.5687** | **ave**  **std** | -0.7567  3.12e-1 | -0.7634  5.75e-4 | -1.7076  0.0560 | -0.3080  0.0864 |
| **=0.0956** | **ave**  **std** | -0.3523  3.36e-6 | -0.3663  4.57e-5 | -1.2727  0.0381 | -0.051  0.0262 | **=0.6161** | **ave**  **std** | -0.7667  3.04e-1 | -0.7867  5.55e-4 | -1.7485  0.0096 | -0.3803  0.1094 |
| **=0.1902** | **ave**  **std** | -0.4021  1.49e-6 | -0.4311  9.82e-5 | -1.3966  0.0296 | -0.08  0.0365 | **=0.6634** | **ave**  **std** | -0.7808  2.65e-1 | -0.8185  3.31e-4 | -1.8494  0.0115 | -0.4047  0.1131 |
| **=0.2375** | **ave**  **std** | -0.4567  1.40e-1 | -0.4822  4.01e-5 | -1.4403  0.0189 | -0.1016  0.0581 | **=0.7107** | **ave**  **std** | -0.8277  2.59e-1 | -0.8577  3.99e-4 | -1.9185  0.0937 | -0.4406  0.0653 |
| **=0.2848** | **ave**  **std** | -0.5078  2.93e-6 | -0.5555  1.18e-4 | -1.4855  0.0303 | -0.1259  0.0805 | **=0.7580** | **ave**  **std** | -0.8556  3.13e-1 | -0.8856  1.76e-3 | -1.8283  0.0761 | -0.5148  0.0357 |
| **=0.3322** | **ave**  **std** | -0.5624  3.44e-6 | -0.5733  3.44e-5 | -1.5457  0.0277 | -0.1381  0.1088 | **=0.8053** | **ave**  **std** | -0.9182  3.80e-1 | -0.9315  3.42e-3 | -1.8710  0.0190 | -0.5429  0.1393 |
| **=0.3795** | **ave**  **std** | -0.5929  2.73e-1 | -0.6211  8.90e-5 | -1.6478  0.0206 | -0.1486  0.1126 | **=0.8526** | **ave**  **std** | -0.9311  4.19e-1 | -0.9711  3.22e-3 | -2.0297  0.0201 | -0.6062  0.1035 |
| **=0.4268** | **ave**  **std** | -0.6545  2.55e-1 | -0.6855  4.36e-4 | -1.5175  0.0597 | -0.1908  0.0412 | **=0.8526** | **ave**  **std** | -0.9843  1.10e-1 | -1.1238  4.78e-3 | -2.0040  0.0469 | -0.6542  0.1390 |
| **=0.4741** | **ave**  **std** | -0.6928  2.44e-1 | -0.7081  2.27e-4 | -1.6995  0.0522 | -0.2453  0.0973 | **=0.9** | **ave**  **std** | -1.0537  9.79e-2 | -1.2631  4.22e-2 | -2.0072  0.0623 | -0.7440  0.1308 |

Table 6 The results of various heuristic algorithms for different payment factor

Table 7 The P-values obtained from (21) under different payment factor and different number of users

| **Aspects** | **BRD-CO V.S**  **PSO. Offl.** | **BRD-CO V.S**  **DE. Offl.** | **BRD-CO V.S**  **SA. Offl.** | **BRD-CO V.S**  **ACO. Offl.** | **BRD-CO V.S**  **L. Offl.** | **BRD-CO V.S**  **F. Offl.** | **BRD-CO V.S**  **G. Offl.** | **BRD-CO V.S**  **R. Offl.** |
| --- | --- | --- | --- | --- | --- | --- | --- | --- |
| **the number of user** | 0.0082 | 0.0071 | 4.86e-5 | 0.0344 | 5.13e-6 | 0.0082 | **0.3325** | 0.231 |
| **payment factor** | 3.39e-8 | 3.38e-8 | 3.39e-8 | 7.99e-6 | 4.01e-9 | 3.36e-8 | 3.37e-8 | 3.35e-8 |
